# Quantifying excess TB prevalence among people living with HIV on ART in the universal ART era: Evidence from a TB prevalence survey in Zambia and South Africa

**DOI:** 10.1371/journal.pgph.0006833

**Published:** 2026-09-21

**Authors:** Thomas Gachie, Eveline Klinkenberg, Kwame Shanaube, Linda Mureithi, Peter J. Dodd, Maria Ruperez, Ab Schaap, Richard Hayes, Helen Ayles, Jonathan Bartlett, Sian Floyd

**Affiliations:** 1 London School of Hygiene & Tropical Medicine (LSHTM), London, United Kingdom; 2 Zambart, Lusaka, Zambia; 3 Sheffield Centre for Health and Related Research, School of Medicine and Population Health, University of Sheffield, Sheffield, United Kingdom; 4 ConnectTB, the Hague, The Netherlands; 5 Health Systems Trust, Cape Town, South Africa; University of California Irvine, UNITED STATES OF AMERICA

## Abstract

Despite widespread antiretroviral therapy (ART) scale-up, tuberculosis (TB) remains a leading cause of morbidity and mortality among people living with HIV (PLHIV). While ART reduces TB incidence among PLHIV, its effect on TB prevalence remains less clear. We estimated the difference in TB prevalence between PLHIV on ART and their counterfactual HIV-negative state, quantifying their proportionate contribution to overall TB prevalence in high-HIV settings across Southern Africa. Using data from the 2019–2021 TREATS TB prevalence survey, a cross-sectional survey of adults aged ≥15 years in 21 communities in Zambia and South Africa (SA) (Western Cape province), we applied Rubin’s “potential outcomes” framework and inverse probability of treatment weighting, with propensity scores estimated using logistic regression to control for measured confounding. We estimated the average treatment effect on the treated (ATT) as the TB prevalence difference (PD) among PLHIV on ART compared to a counterfactual scenario where the same individuals were HIV-negative. Among 43,617 analysed, 6,612 (15.2%) were PLHIV on ART and 37,005 (84.8%) tested HIV-negative. After adjusting for confounding, PLHIV on ART had higher TB prevalence than the counterfactual HIV-negative scenario (ATT: PD = 0.53%; 95% confidence interval [CI]: 0.19, 0.87). There was statistical evidence of an effect in the Zambian communities (ATT: PD = 0.49%; 95% CI: 0.14, 0.85), but the evidence was weaker in SA communities (ATT: PD = 0.56%; 95% CI: -0.15, 1.26). At the population level, PLHIV on ART contributed an absolute excess of 0.08% to overall TB prevalence, representing a 9.3% proportionate increase, which was larger in Zambian (14.8%) than in SA (5.6%) communities due to background TB prevalence differences. PLHIV on ART remain at elevated risk of prevalent TB, contributing considerably to overall TB prevalence, underscoring the need for continued, targeted TB prevention, screening, and treatment strategies for all PLHIV in settings with a high burden of both TB and HIV.

Trial registration

ClinicalTrials.gov NCT03739736

## Introduction

Tuberculosis (TB) remains a major global health challenge, with an estimated 10.7 million new (incident) cases and 1.23 million TB-related deaths in 2024. Of these deaths, 150,000 occurred among people living with HIV (PLHIV) and 1.08 million among HIV-negative individuals [1]. HIV substantially increases the risk of progression from being infected with *Mycobacterium tuberculosis* (MTB) to developing TB disease [2]. This interaction has shaped the trajectory of both epidemics, especially in countries in Sub-Saharan Africa (SSA) where HIV prevalence is high.

Global strategies have set ambitious targets to reduce the dual burden of TB and HIV. The World Health Organization (WHO) End TB Strategy aims to reduce TB incidence by 90% and TB deaths by 95%, and eliminate catastrophic costs by 2035 [3,4]. The United Nations (UN) Sustainable Development Goal 3.3 calls for ending the epidemics of HIV and TB by 2030 [5]. Complementing these, the Joint UN Programme on HIV/AIDS (UNAIDS) established the 90-90-90 targets in 2014 [6], and more recently the 95-95-95 targets for 2030, aiming for 95% of PLHIV to know their HIV status, 95% of those diagnosed to receive antiretroviral therapy (ART), and 95% of those on ART to achieve viral suppression [7]. Together, these strategies highlight ART as a cornerstone intervention for reducing mortality and HIV-associated TB.

Universal ART initiation - recommended by WHO since 2015 [8] - has been strongly associated with declines in TB incidence and mortality across SSA [9–11]. Community-wide declines in TB transmission have been demonstrated in both trial and population-based studies [10,11]. However, while universal ART is highly effective in reducing TB incidence, its effect on TB prevalence is more complex. This is because TB prevalence reflects both the incidence rate of new TB disease and also the duration of untreated illness, including sub-clinical disease that may not be detected through routine symptom-based screening. Excess prevalent TB may persist due to a combination of factors, including incomplete immune recovery, delayed TB diagnosis or treatment initiation, atypical TB symptoms, and other barriers to timely detection [12–14].

TB prevalence provides a policy-relevant measure of the current burden of TB disease, and understanding whether this burden differs between PLHIV receiving ART and HIV-negative individuals is particularly relevant in the universal ART era. Regular engagement with HIV care should provide PLHIV receiving ART with repeated opportunities for TB screening and diagnosis and referral for TB treatment. However, this routine clinical screening is usually based only on TB symptoms, and so it misses asymptomatic TB. TB prevalence surveys, on the other hand, use both symptom screening and chest X-ray screening; this means they can identify asymptomatic disease [15], including TB that has not been diagnosed despite regular healthcare contact.

The TREATS TB prevalence survey was conducted in 21 communities in Zambia and the Western Cape Province of South Africa (SA) during 2019–2021, and included around 50,000 survey participants aged ≥15 years. Previous analyses of these data found a higher TB prevalence among PLHIV than among HIV-negative individuals, overall and also when restricting the PLHIV population to those who self-reported they were on ART (i.e., excluding individuals who were newly diagnosed with HIV on the day of the survey, and PLHIV who self-reported they were living with HIV and not on ART) [16]. The analyses presented here add to and extend the previous work in several ways: they estimate TB prevalence differences rather than odds ratios, they control more completely for confounding (previous analyses adjusted for the most important confounding variables [community, sex, and age group] but not for others [such as socio-economic position]), and they formally define the target estimand within a “potential outcomes” framework [17].

In the analyses presented here, we applied the Rubin’s potential outcomes framework to estimate the excess prevalence of TB within the population of PLHIV who were on ART. Within this framework, the exposure was defined as the composite state of being an individual living with HIV and on ART, compared with being HIV-negative. This approach allowed us to compare the observed prevalence of TB among PLHIV on ART, with the counterfactual scenario in which those same individuals were HIV-negative (the average treatment effect on the treated, ATT). By reporting prevalence differences rather than relative measures, we aimed to quantify the excess burden of prevalent TB among PLHIV on ART in absolute terms, after adjustment for measured confounding variables.

## Materials and Methods

### Ethics statement

The TREATS study received ethical approval from the London School of Hygiene & Tropical Medicine (LSHTM), the University of Zambia Biomedical Research Ethics Committee (UNZABREC) and the National Health Research Authority (NHRA), and Pharma-Ethics (Pty), South Africa. Written informed consent was obtained from participants aged ≥18 years, and assent with parental consent from those aged 15–17 years. The study was registered at ClinicalTrials.gov (Identifier: NCT03739736; registered on 14/10/2018). Additional ethical approval for the secondary analyses reported in this manuscript was granted by the LSHTM Research Ethics Committee on 10/07/2023. Data were accessed for research purposes on 10/07/2023. The authors did not have access to information that could identify individual participants during or after data collection.

### Study design and setting

The TREATS TB prevalence survey was a cross-sectional survey that was carried out during 2019–2021, before and during the COVID-19 pandemic [16,18,19]. Data collection was conducted across 12 communities in Zambia (02 February 2019 to 04 July 2021) and 9 communities in the Western Cape Province of SA (25 March 2020 to 23 June 2021) that were included in the HPTN 071 (PopART) trial [20,21].

The primary aim of the survey was to estimate the prevalence of bacteriologically confirmed pulmonary TB in a random sample of approximately 50,000 individuals aged ≥15-years, and compare TB prevalence among the three arms of the HPTN 071 (PopART) trial. Full details of the sampling, recruitment, and diagnostic procedures (symptom screening, chest x-ray, Xpert-Ultra, and culture), are detailed elsewhere [16,18,20]. Briefly, eligible individuals attended a mobile field site where they completed questionnaires on socio-demographic and socio-economic characteristics, behavioural risk factors, and HIV-related history, were screened for TB symptoms, and underwent chest X-ray examination. Chest X-rays were captured digitally, and computer-assisted-diagnosis (CAD) was used to score each X-ray between 0 and 100 (with a higher score indicating a higher probability of having prevalent TB). Individuals who screened positive on TB symptoms, or had an X-ray CAD score ≥50, were asked to give two sputum samples for TB diagnosis using Xpert-Ultra testing (with one or two additional sputum samples collected for culture testing, for individuals with weakly positive or discordant Xpert-Ultra test results).

### Outcome definition

The outcome variable was prevalent TB, defined as bacteriologically confirmed pulmonary TB based on Xpert-Ultra and culture testing. Individuals who screened positive by symptoms or X-ray but lacked bacteriological test results had prevalent TB status imputed. Individuals who screened negative by symptoms and X-ray were assumed to not have prevalent TB. Further details of the imputation approach for missing data on prevalent TB, and on the outcome definition have been provided elsewhere [16].

### Exposure definition and comparison group

The primary exposure of interest was defined as a joint, composite state of being an individual living with HIV and currently on ART. During the survey HIV interview, participants reported their HIV testing history, most recent HIV test result, and current ART use (yes/no). HIV testing was offered to all participants who did not self-report living with HIV.

For our analysis, we evaluated the contrast between PLHIV on ART (self-reported) against those who tested HIV-negative. Participants who were newly diagnosed as HIV-positive during the survey, living with HIV but not on ART, or who did not accept the offer of HIV testing, were not included in analysis (See Fig 2).

### Research question, causal framework and estimands

We aimed to quantify the excess TB prevalence among PLHIV on ART, compared with the counterfactual scenario in which those same individuals were HIV-negative. Our analysis was done using Rubin’s “potential outcomes” framework [17]. Within this framework, the composite state of being an individual living with HIV and on ART defined the treated group, while being HIV-negative defined the untreated group. This approach allowed us to estimate the counterfactual prevalence of TB that would have been observed among PLHIV on ART had those same individuals been HIV-negative.

Our primary objective was to estimate, among PLHIV on ART, the difference in prevalent TB compared with the counterfactual scenario in which those same individuals were HIV-negative; this contrast is referred to as the average treatment effect on the treated (ATT). As a secondary objective, we estimated the average treatment effect (ATE), which compares the counterfactual scenario in which the entire population is “treated” (living with HIV and on ART) with the counterfactual scenario in which the entire population is “untreated” (HIV-negative). While the ATE provides a population-wide contrast, the scenario that all individuals in a population are PLHIV is not realistic. The ATT is a more useful and policy-relevant comparison, because it quantifies the excess TB prevalence among PLHIV who are engaged in ART programmes.

We chose to estimate and report prevalence differences (PDs) rather than odds ratios or risk ratios. In addition to being directly interpretable for quantifying the excess burden of TB [22], PDs align naturally with additive marginal contrasts that are defined under the potential outcomes framework [23]. This measure is valuable for informing public health action and identifying the scale of the diagnostic gap in clinical settings in absolute terms [22].

### Causal assumptions

To estimate these causal effects from our observational data, several assumptions were required. First, “*no interference”,* meaning that one individual’s exposure does not influence another’s outcome. Although this assumption may be violated in infectious disease settings because ART use may indirectly influence TB transmission within communities, we assumed that such spillover effects were unlikely to materially affect estimation of individual-level prevalent TB at the time of the survey. Second, “*consistency”,* meaning that exposure categories are well defined across all individuals (and that observed outcomes under a given exposure level are equal to their corresponding “potential outcome”). Third, “*conditional exchangeability”*, meaning that after we adjust for a minimally sufficient set of measured confounding variables, the treated and untreated groups are comparable (this is the no unmeasured confounding assumption). Fourth, “*positivity”,* meaning that for every combination of measured confounding variables, there is a non-zero probability of an individual being in either the treated or untreated group. Fifth, “*correct model specification”,* meaning that the statistical model used to adjust for confounding includes all confounding variables and is correctly specified. Further discussion is provided in the supplementary section (See Text D in S1 Appendix). In addition to the standard causal assumptions, we also assumed that the exposure preceded the onset of the outcome. We acknowledge that a cross-sectional survey design does not allow definitive establishment of the temporal sequence between HIV/ART status and prevalent TB. However, we assumed that the composite exposure state - being an individual living with HIV on ART - preceded the detection of prevalent TB on the day of the survey.

### Controlling for confounding variables

**Fig 1:**
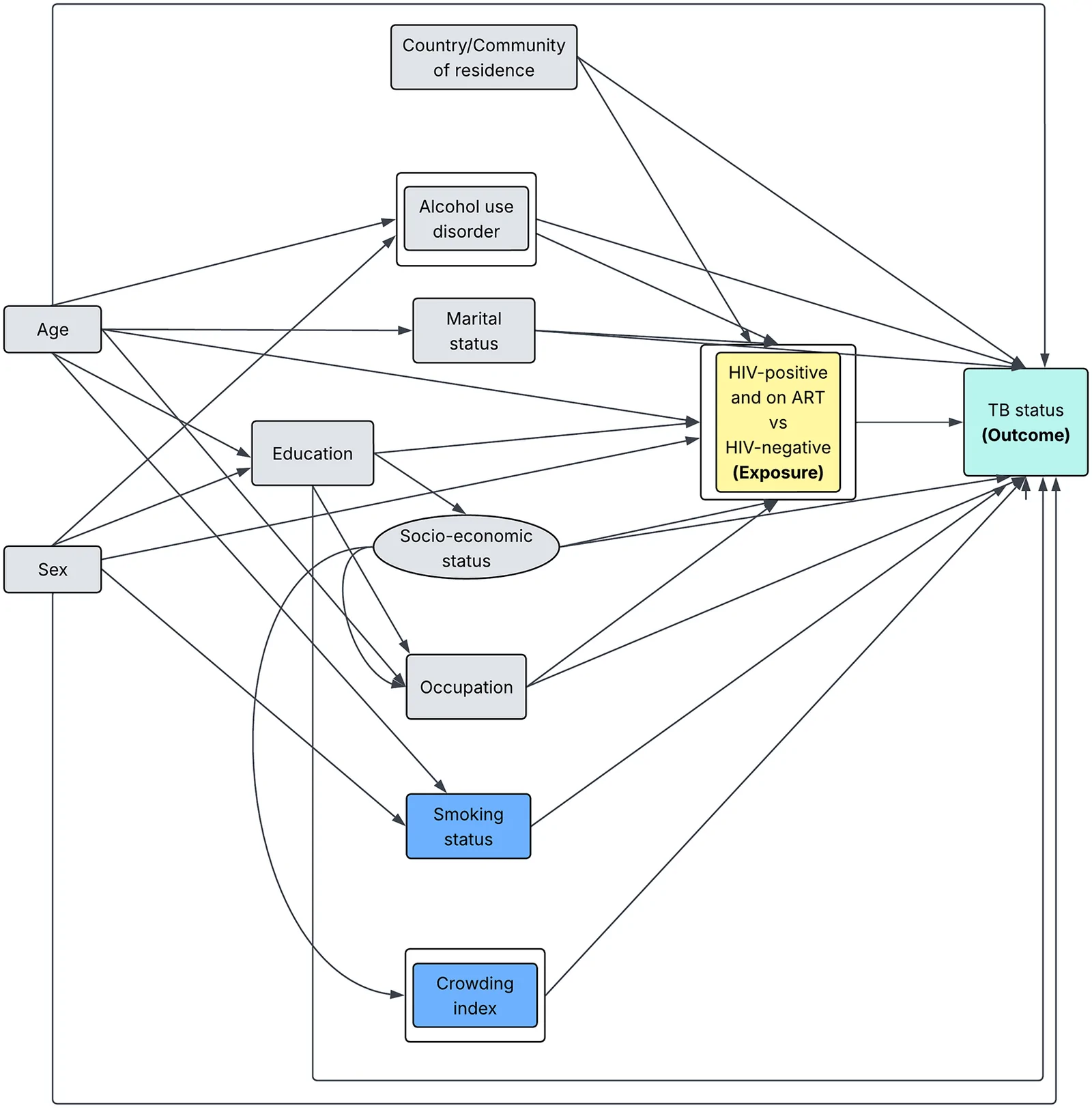
Directed acyclic graph showing assumed causal relationships between HIV/ART status and prevalent tuberculosis. Rectangles denote observed variables; double-outlined rectangles represent composite or derived clinical and structural variables (exposure, alcohol use disorder, crowding index) as recommended by Berrie et al [26]. The ellipse represents the latent socio-economic status construct using structural equation modelling notation, as suggested by Franklin et al [27]. Grey nodes represent demographic, socio-economic, and behavioural covariates. Blue nodes represent the predictors of TB. Yellow indicates the primary composite exposure (“living with HIV and on ART compared with being HIV-negative”) and green indicates the outcome (TB status). Arrows denote hypothesised causal relationships used to guide confounder selection.

A directed acyclic graph (DAG) was constructed using domain knowledge to represent the assumed causal relationships between potential confounders, HIV/ART status and prevalent TB. The causal structure was specified in DAGitty software [24], and the diagram was reformatted in Lucidchart [25] for presentation. The DAG was used to guide identification of a minimally sufficient adjustment set of confounders. Our DAG indicated that the following variables required adjustment: age group (with categories determined *a priori* as 15–19, 20–24, 25–29, 30–39, 40–49, 50–59, ≥ 60 years), sex, country and community of residence, marital status, education (grouped as none, primary, secondary, higher), occupation (grouped as unemployed, occasional/seasonal employment, government employment, private sector employment, self-employed, student, housewife/homemaker, working on own land, other), risk of alcohol use disorder (low risk, medium risk, high risk, addiction likely), and household socio-economic position (SES) (lowest, second, middle, fourth, highest quintile). Variables believed to only affect the outcome (and not HIV/ART status), such as smoking (never-, current-, past- smoker) and crowding index, were also included in the DAG (See Fig 1). The complete epidemiological and structural justification for this chosen adjustment set is detailed in the supplementary section (See Text D in S1 Appendix).

SES was estimated separately for Zambia and SA, using principal components analysis based on household asset ownership. The risk of alcohol use disorder was assessed at the individual level using the WHO AUDIT screening tool [28,29], while crowding index was calculated as the number of household members divided by the number of sleeping rooms. Further details on the definitions and construction of these variables are given in the appendix (See Texts A - C in S1 Appendix).

### Statistical analysis

To adjust for confounding, we applied the method of inverse probability of treatment weighting (IPTW) [30,31], based on estimated propensity scores (PS), i.e., the predicted probability of being an individual living with HIV and on ART given an individual’s covariates. Propensity scores (PS) were estimated using logistic regression, with the outcome variable being whether an individual was living with HIV and on ART (yes or no) (Table C in S1 Appendix). Explanatory variables were pre-specified confounders (based on the DAG) and predictors of prevalent TB, with interaction terms (age group and sex, sex and country, and age group and country) also included based on prior knowledge (See more detail in Text D in S1 Appendix).

IPTW reweights all individuals to create a pseudo-population in which the distribution of measured covariates is balanced between treated and untreated groups, thus controlling for confounding. We used unstabilised IPTWs for both the ATT and ATE analyses [32,33]. For the ATT, individuals in the treated group (PLHIV on ART) were assigned a weight of one, and HIV-negative individuals were reweighted using the odds of being in the treated group (PS/[1–PS]) to align their covariate distribution with that of the treated group. For the ATE, treated individuals were weighted by 1/PS and untreated individuals by 1/(1–PS) [31].

Some causal assumptions were assessed using diagnostic checks. Positivity was evaluated by examining the overlap of propensity score distributions between the treated and untreated groups, with kernel density plots. Covariate balance was assessed by comparing absolute standardized mean differences (ASMD) before and after weighting, with values <10% considered to indicate adequate balance. Balance was also visualized using love plots generated with the cobalt package in R [34]. E-values were used to assess the robustness of our findings to potential unmeasured confounding [35,36] (See more detail in Text G in S1 Appendix).

Prevalence differences were estimated for both the primary objective (ATT) and secondary objective (ATE), with the findings of the latter detailed in supplementary material (Text F in S1 Appendix).

To estimate the population-wide effect on TB prevalence of being an individual living with HIV and on ART versus being HIV negative, we multiplied the estimated excess prevalent TB among PLHIV on ART (i.e., ATT) by the proportion of the population who were PLHIV on ART, yielding the absolute excess TB prevalence attributable to this group. The proportionate contribution of PLHIV on ART to overall prevalent TB was then calculated as excess prevalent TB divided by the observed overall prevalence of TB.

Analyses were repeated for prespecified sub-groups [37], defined by country (Zambia or SA), sex (male or female), age (younger [<30 years] or older [≥30 years]), and COVID-19 period (before or during the pandemic). All analyses were conducted on 300 imputed datasets, previously generated for the primary analysis of the TB prevalence survey [16], with estimates pooled using Rubin’s rules [38]. Statistical analyses were performed in R (version 4.4.2) [39] and Stata (version 19.5) [40].

## Results

### Characteristics of the study population

**Fig 2:**
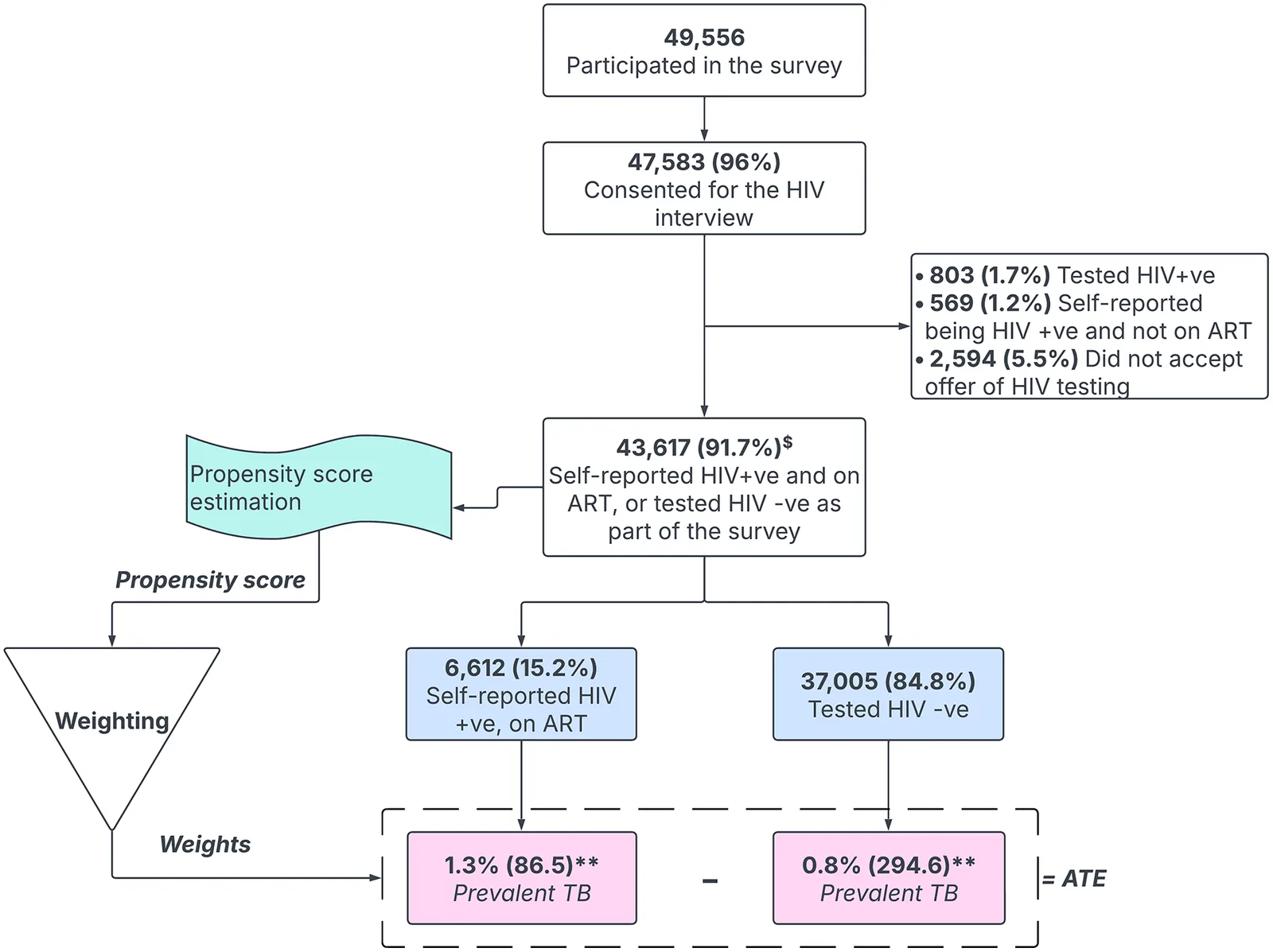
Study flow diagram and analytic sample used for propensity score–weighted analysis. ^$^Data used in the propensity score estimation; **Imputed outcome data was used in the primary analysis (Klinkenberg and Floyd et al., 2023 [16]); HIV, Human Immunodeficiency Virus; ART, Antiretroviral therapy; TB, Tuberculosis; Prevalent TB (defined as bacteriologically confirmed pulmonary TB based on Xpert-Ultra or culture testing); TB prevalence was 2.5% (estimated n = 19.9 /803) among individuals newly diagnosed as HIV-positive, 1.6% (estimated n = 9.0/569) among those who self-reported living with HIV and not on ART, and 0.9% (estimated n = 23.9/2,594) among those who did not accept the offer of HIV testing.

Among 29,275 randomly selected households, 49,556 individuals consented to participate in the survey; 47,583 (96%) consented to the HIV interview and self-reported their current HIV status. After excluding 803 (1.7%) individuals newly diagnosed as HIV-positive during the survey, 569 (1.2%) who self-reported living with HIV and that they were not on ART, and 2,594 (5.5%) who did not accept the offer of HIV testing, 43,617 participants were included in analysis. Of these, 6,612 (15.2%) were PLHIV on ART, while 37,005 (84.8%) tested HIV-negative (Fig 2, Table 1).

**Table 1:** Baseline characteristics of survey participants stratified by HIV status.

		HIV Status		
**Potential list of confounders**	**Overall**	**HIV Negative**	**PLHIV, on ART**	P-value*
	**43,617**	**37,005**	**6,612**	
	**n (%)**	**n (%)**	**n (%)**	
**Country**				
*Zambia*	27,827 (63.8%)	23,630 (63.9%)	4,197 (63.5%)	
*South Africa*	15,790 (36.2%)	13,375 (36.1%)	2,415 (36.5%)	0.6
**Community of residence**				
*community 1*	1,735 (4.0%)	1,516 (4.1%)	219 (3.3%)	
*community 2*	1,831 (4.2%)	1,579 (4.3%)	252 (3.8%)	
*community 3*	3,180 (7.3%)	2,742 (7.4%)	438 (6.6%)	
*community 4*	3,219 (7.4%)	2,673 (7.2%)	546 (8.3%)	
*community 5*	1,900 (4.4%)	1,654 (4.5%)	246 (3.7%)	
*community 6*	1,927 (4.4%)	1,638 (4.4%)	289 (4.4%)	
*community 7*	3,428 (7.9%)	2,949 (8.0%)	479 (7.2%)	
*community 8*	2,692 (6.2%)	2,322 (6.3%)	370 (5.6%)	
*community 9*	1,972 (4.5%)	1,666 (4.5%)	306 (4.6%)	
*community 10*	1,557 (3.6%)	1,180 (3.2%)	377 (5.7%)	
*community 11*	1,485 (3.4%)	1,243 (3.4%)	242 (3.7%)	
*community 12*	2,901 (6.7%)	2,468 (6.7%)	433 (6.5%)	
*community 13*	1,637 (3.8%)	1,346 (3.6%)	291 (4.4%)	
*community 14*	1,920 (4.4%)	1,568 (4.2%)	352 (5.3%)	
*community 15*	3,572 (8.2%)	2,836 (7.7%)	736 (11.1%)	
*community 16*	1,613 (3.7%)	1,295 (3.5%)	318 (4.8%)	
*community 17*	2,461 (5.6%)	1,960 (5.3%)	501 (7.6%)	
*community 18*	589 (1.4%)	511 (1.4%)	78 (1.2%)	
*community 19*	1,137 (2.6%)	1,087 (2.9%)	50 (0.8%)	
*community 20*	1,054 (2.4%)	1,003 (2.7%)	51 (0.8%)	
*community 21*	1,807 (4.1%)	1,769 (4.8%)	38 (0.6%)	<0.001
**Age category**				
*15-19*	8,536 (19.6%)	8,382 (22.7%)	154 (2.3%)	
*20-24*	7,746 (17.8%)	7,440 (20.1%)	306 (4.6%)	
*25-29*	6,445 (14.8%)	5,702 (15.4%)	743 (11.2%)	
*30-39*	9,148 (21.0%)	6,818 (18.4%)	2,330 (35.2%)	
*40-49*	5,558 (12.7%)	3,582 (9.7%)	1,976 (29.9%)	
*50-59*	3,353 (7.7%)	2,550 (6.9%)	803 (12.1%)	
*60+*	2,831 (6.5%)	2,531 (6.8%)	300 (4.5%)	<0.001
**Sex**				
*Male*	16,876 (38.7%)	15,335 (41.4%)	1,541 (23.3%)	
*Female*	26,741 (61.3%)	21,670 (58.6%)	5,071 (76.7%)	<0.001
**Highest level of education completed**				
*None*	1,332 (3.1%)	1,110 (3.0%)	222 (3.4%)	
*Primary*	9,546 (21.9%)	7,802 (21.1%)	1,744 (26.4%)	
*Secondary*	30,666 (70.3%)	26,243 (70.9%)	4,423 (66.9%)	
*Higher*	2,073 (4.8%)	1,850 (5.0%)	223 (3.4%)	<0.001
**Socio-economic status** ^**‡**^				
*Lowest*	8,645 (19.8%)	7,159 (19.3%)	1,486 (22.5%)	
*Second*	9,144 (21.0%)	7,693 (20.8%)	1,451 (21.9%)	
*Middle*	8,753 (20.1%)	7,457 (20.2%)	1,296 (19.6%)	
*Fourth*	7,787 (17.9%)	6,682 (18.1%)	1,105 (16.7%)	
*Highest*	9,288 (21.3%)	8,014 (21.7%)	1,274 (19.3%)	<0.001
**Marital status**				
*Never married*	24,451 (56.1%)	21,924 (59.2%)	2,527 (38.2%)	
*Currently married or living as married*	14,655 (33.6%)	11,923 (32.2%)	2,732 (41.3%)	
*Divorced or separated*	2,491 (5.7%)	1,779 (4.8%)	712 (10.8%)	
*Widowed*	2,020 (4.6%)	1,379 (3.7%)	641 (9.7%)	<0.001
**Alcohol use disorder** ^**§**^				
*Low risk:0–7*	37,204 (85.3%)	31,838 (86.0%)	5,366 (81.2%)	
*Medium risk:8–15*	4,186 (9.6%)	3,385 (9.1%)	801 (12.1%)	
*High risk:16–19*	1,080 (2.5%)	878 (2.4%)	202 (3.1%)	
*Addiction likely:20–40*	1,147 (2.6%)	904 (2.4%)	243 (3.7%)	<0.001
**Occupation**				
*Unemployed*	19,242 (44.1%)	16,194 (43.8%)	3,048 (46.1%)	
*Occasional/seasonal employment*	1,454 (3.3%)	1,204 (3.3%)	250 (3.8%)	
*Employed by government*	809 (1.9%)	675 (1.8%)	134 (2.0%)	
*Employed in private sector*	4,961 (11.4%)	4,146 (11.2%)	815 (12.3%)	
*Self-employed*	7,226 (16.6%)	5,662 (15.3%)	1,564 (23.7%)	
*Pupil / student*	6,450 (14.8%)	6,332 (17.1%)	118 (1.8%)	
*Housewife / homemaker*	2,629 (6.0%)	2,127 (5.7%)	502 (7.6%)	
*Working on own land*	178 (0.4%)	155 (0.4%)	23 (0.3%)	
*Other*	668 (1.5%)	510 (1.4%)	158 (2.4%)	<0.001
**Crowding index** ^**†**^				
*Mean (SD)*	1.72 (1.1)	1.73 (1.1)	1.72 (1.2)	0.3**
**Smoking status**				
*Never smoked*	31,957 (73.3%)	26,838 (72.5%)	5,119 (77.4%)	
*Current smoker*	9,425 (21.6%)	8,280 (22.4%)	1,145 (17.3%)	
*Past smoker*	2,235 (5.1%)	1,887 (5.1%)	348 (5.3%)	<0.001
***Main exposure (HIV status)***				
*Tested HIV-negative (on the day of the survey)*	37,005 (84.8%)	–	–	
*PLHIV, on ART*	6,612 (15.2%)	–	–	

Note: ‡ Socio-economic status = Estimated from household asset ownership (separately for Zambia and SA) using Principal Components Analysis, with the first principal component categorized into quintiles. Further details in the appendix (Text A in S1 Appendix). § Alcohol use disorder = Scored and classified according to the WHO AUDIT screening tool (Saunders et al., 1993). Further details in the appendix (Text B in S1 Appendix). †Crowding index = number of people per room in a household. Further details on the distribution are in the appendix (Text C in S1 Appendix). *P-value = from a chi-square test; **P-value = from an unpaired t-test; SD = Standard deviation; %Column percentages; PLHIV = People living with HIV

Most participants were from Zambia (64%), female (61%), under 30 years old (52%), had completed secondary education (70%), and had never been married before (56%). Around 5% were classified as being at a high risk of alcohol use disorder, 27% self-reported they were current or past smokers, 44% were unemployed, and the mean household crowding index was 1.7 individuals aged ≥15 years per sleeping room (Table 1).

The overall proportion of individuals who were PLHIV on ART was 15.2%, similar in Zambia (15.1%) and SA (15.3%). These proportions were higher among females (19.0%) than males (9.1%), and among older [>30 years] (25.9%) compared to younger [<30 years] individuals (5.3%). Communities surveyed before the COVID-19 pandemic had slightly higher proportions of PLHIV on ART (mean of 15.9%) than those surveyed during the pandemic (mean of 14.4%).

TB prevalence was 0.7% (302) in the complete case analysis, and estimated as 0.9% (estimated n = 381.1) after multiple imputation of missing data on prevalent TB. Using the imputed datasets, prevalence of TB was higher in SA (1.5%, estimated n = 241.1) than in Zambia (0.5%, estimated n = 140.0), higher among PLHIV on ART (1.3%, estimated n = 86.5) than among HIV-negative individuals (0.8%, estimated n = 294.6), and higher among men (1.5%, estimated n = 248.8) than in women (0.5%, estimated n = 132.3) (Table B in S1 Appendix).

### Overlap and covariate balance

**Fig 3:**
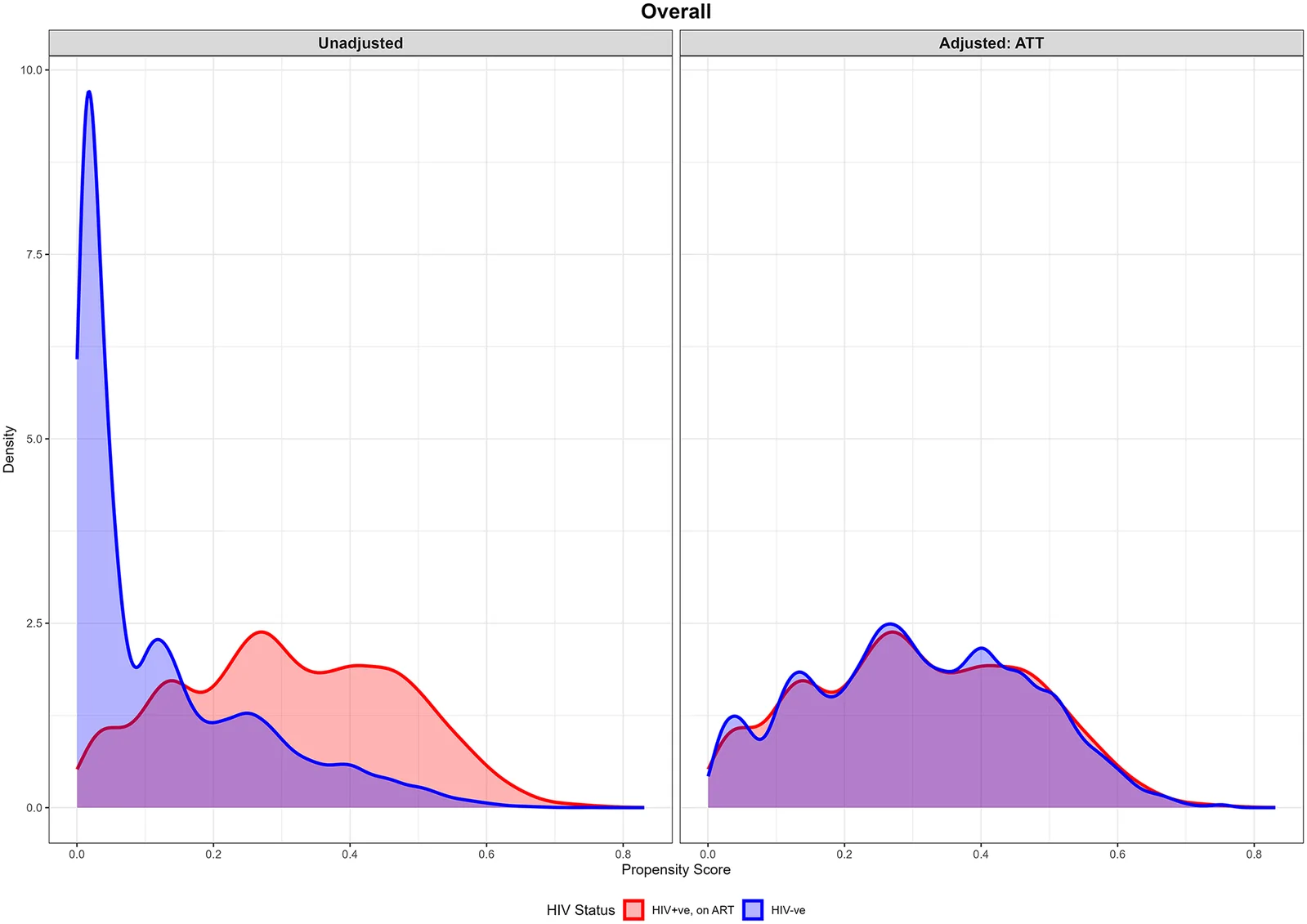
Overall propensity score distributions before and after weighting for HIV status groups. Density plots showing the overall distribution of estimated propensity scores among PLHIV on ART and HIV-negative individuals before weighting and after inverse probability weighting for the average treatment effect among the treated (ATT). Improved overlap after weighting indicates improved covariate balance in the overall analytic population.

Propensity score distributions demonstrated high overlap between exposure groups across all strata (Fig 3, Figs L- P in S1 Appendix). Before weighting, imbalances (ASMD > 10%) were observed for community, sex, age group, marital status, and smoking (Figs I - J in S1 Appendix). After applying IPTW, covariate balance improved substantially for both the ATT and ATE analyses, with only small residual imbalances in a few subgroups (Fig K in S1 Appendix). Weight diagnostics showed generally stable ATT weights with limited evidence of extreme weighting, while ATE weights demonstrated greater variability but retained substantial overlap between exposure groups (Text E; Table D; Figs Q - R in S1 Appendix).

### Overall analysis, ATT

**Fig 4:**
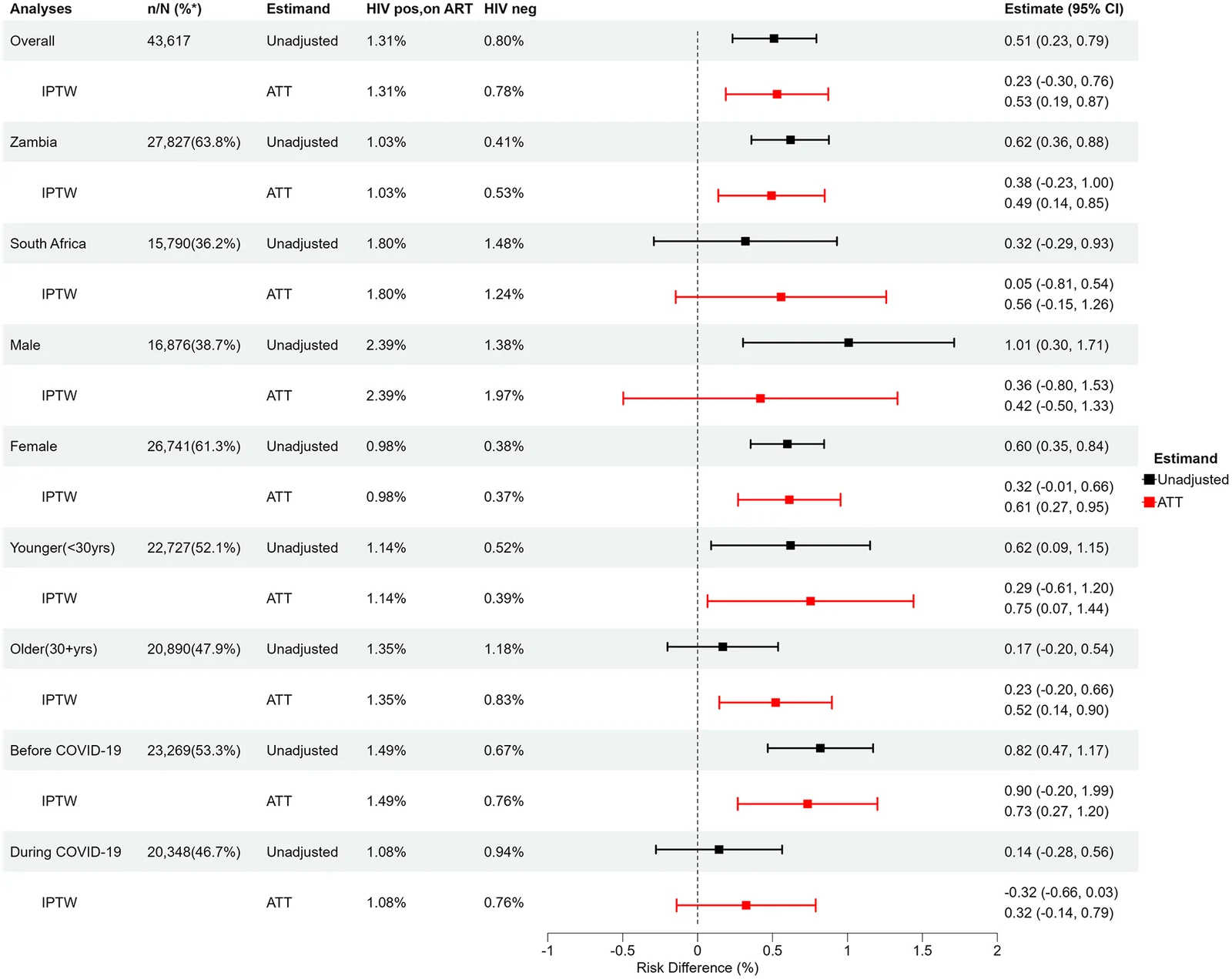
TB Prevalence difference estimates: overall and by subgroup. Forest plot showing unadjusted and IPTW–adjusted (ATT) prevalence differences in comparing PLHIV on ART with HIV-negative individuals. These are presented overall and also stratified by country, sex, age group, and COVID-19 period. Points represent prevalence difference estimates and horizontal lines indicate 95% CIs. ATT, average treatment effect among the treated; HIV pos, Individual living with HIV; HIV neg, HIV negative; CI, Confidence interval; n/N%* (N = 43,617); IPTW, Inverse probability of treatment weighting.

In the unadjusted analysis, TB prevalence was higher among PLHIV on ART compared to HIV-negative individuals (Prevalence difference [PD] = 0.51% (95% Confidence interval [CI]: 0.23,0.79). After adjusting for confounding, the estimated ATT PD was 0.53% (95% CI: 0.19, 0.87), comparing the observed prevalence among PLHIV on ART to the counterfactual scenario in which those same individuals had been HIV-negative (Fig 4).

### Subgroup analyses, ATT

Stratified ATT estimates consistently indicated higher TB prevalence among PLHIV on ART compared with their counterfactual HIV-negative states across all evaluated subgroups, with some variations in the estimated magnitude of effect (Fig 4).

In Zambian communities, the adjusted PD was 0.49% (95% CI: 0.14, 0.85), comparing PLHIV on ART to their counterfactual HIV-negative state. In South African communities, the estimated PD was 0.56% (95% CI: -0.15, 1.26) under the same comparison. Sex-specific analyses showed an adjusted PD of 0.61% (95% CI: 0.27, 0.95) among female PLHIV on ART compared with the counterfactual scenario in which they were HIV-negative. Among males, the estimated PD between the observed and counterfactual states was 0.42% (95% CI: −0.50, 1.33). Age-stratified analyses showed a PD of 0.75% (95% CI: 0.07, 1.44) among younger PLHIV on ART (<30 years) compared to their counterfactual HIV-negative state, and 0.52% (95% CI: 0.14, 0.90) among older (>30 years) individuals under the same counterfactual contrast. When stratified by survey timeline, the estimated PD was 0.73% (95% CI: 0.27, 1.20) prior to the COVID-19 pandemic and 0.32% (95% CI: −0.14, 0.79) during the pandemic period, comparing PLHIV on ART to their counterfactual HIV-negative state.

### Estimated contribution of excess TB prevalence among PLHIV on ART to overall TB prevalence

At the population level, with the prevalence of PLHIV on ART being 15.2%, the ATT estimate (0.53%) translated into an absolute value of excess TB prevalence of 0.081%, equivalent to a 9.3% proportionate contribution to the overall TB prevalence in the total study population (i.e., PLHIV on ART and HIV-negative individuals). In Zambian communities (background TB prevalence 0.50%), the excess was 0.074% (14.8% relative increase). In SA communities (background TB prevalence 1.53%), the excess was slightly higher at 0.086%, but due to the higher overall TB prevalence this represented a smaller relative increase of 5.6% (Table 2).

**Table 2:** Estimated excess TB prevalence attributable to PLHIV on ART.

	ATT (% TB prevalence difference)	Prevalence of PLHIV on ART (%)	Overall TB prevalence‡ (%)	Absolute excess TB prevalence (%) *	Proportionate increase in TB prevalence (%) ^§^
Overall	0.53	15.2	0.87	0.081	9.3
Zambia	0.49	15.1	0.50	0.074	14.8
South Africa	0.56	15.3	1.53	0.086	5.6

Note: PLHIV = People living with HIV; ATT = Average treatment effect amongs the treated (PLHIV on ART); *AbsoluteexcessTBprevalence=ATTx%prevalenceofHIV−positiveonART(indecimalform); ^§^ $ProportionateincreaseinTBprevalence=\frac{ExcessTBprevalence}{OverallTBprevalence}x\text{1}\text{0}\text{0}$; ‡ Observed TB prevalence for both PLHIV on ART and HIV-negative

### ATE analyses

For comparison, the adjusted ATE showed an estimated PD of 0.23% (95% CI: -0.30, 0.73), contrasting the counterfactual scenario in which all individuals were PLHIV on ART versus the counterfactual scenario in which all individuals were HIV-negative [ATE: PD = 0.23% (95% CI: -0.30, 0.73)]. Among females, this counterfactual contrast yielded an ATE prevalence difference of 0.32% (95% CI: -0.01, 0.66). In communities surveyed during the COVID-19 pandemic, the ATE PD under the same counterfactual comparison was -0.32% (95% CI: -0.66, 0.03) (Text F; Fig S in S1 Appendix).

## Discussion

Our study found evidence of persistent excess TB prevalence among PLHIV on ART compared to the counterfactual scenario in which those same individuals were HIV-negative. Our primary estimand - the average treatment effect on the treated (ATT) - showed an estimated overall prevalence difference (PD) of 0.53% (95% CI: 0.19, 0.87), across communities where the observed TB prevalence among PLHIV on ART was around 1.3%. This indicates that even in the universal ART era, a considerable excess burden of prevalent TB persists within this population. Crucially, because TB prevalence reflects both disease occurrence and duration, these findings suggest ongoing excess risk of developing incident TB among PLHIV who are on ART (compared with HIV-negative individuals) or persistent gaps in TB detection and care among PLHIV who are engaged in routine ART programmes. This excess was consistent across settings, with an ATT PD of 0.49% (95% CI: 0.14, 0.85) in Zambian communities and 0.56% (95% CI: −0.15, 1.26) in SA communities. Together, these findings indicate that PLHIV on ART continue to experience higher TB prevalence compared with HIV-negative individuals, though the magnitude of excess in relative terms varied by setting.

These findings are consistent with earlier TREATS analyses that adjusted for confounding by community, age group, and sex and used odds ratios as a relative measure of association between HIV status and TB prevalence [16]. Those earlier analyses reported higher TB prevalence among PLHIV compared with HIV-negative individuals in both Zambia and SA, with adjusted odds ratios (AORs) of 2.29 (95% CI: 1.54, 3.41) and 1.61 (95% CI: 1.13, 2.30), respectively; when comparing PLHIV on ART with HIV-negative individuals the AORs were 2.36 (95% CI: 1.55, 3.57) and 1.50 (95% CI: 1.01, 2.34) respectively. The analyses presented here add to and extend the earlier analyses by focusing specifically on PLHIV on ART, by applying a formal causal inference framework, and controlling for a broader set of confounding variables identified through a DAG. Expressing this causal contrast as a prevalence difference provides a direct estimate of the excess TB prevalence among PLHIV on ART in absolute terms. Our findings are also consistent with other population-based TB prevalence surveys conducted in high HIV-burden settings in the universal ART era, which have repeatedly shown higher TB prevalence among PLHIV than among HIV-negative individuals. A community-based survey in urban Malawi (2019–2020) estimated TB prevalence at 292 per 100,000 among PLHIV compared with 148 per 100,000 among HIV-negative individuals [41]; this study also conducted adjusted analyses, although the number of TB cases among PLHIV was small, limiting precision. In contrast, the SA national TB prevalence survey reported descriptive results only, showing that 28.8% of prevalent TB cases occurred among PLHIV despite widespread ART coverage [42]. However, these surveys primarily compared TB burden by HIV status and did not stratify PLHIV according to ART use. By directly comparing the observed prevalence of TB among PLHIV on ART with the counterfactual scenario in which those same individuals were HIV-negative, adjusting for measured confounding variables with a potential outcomes framework, our analysis demonstrates that an excess prevalent TB burden persists among PLHIV even among individuals who are on ART.

The proportional contribution of PLHIV on ART to the overall community-wide TB burden depended on the background TB prevalence. In Zambian communities, where the overall TB prevalence was around 0.5%, the excess TB prevalence among PLHIV on ART contributed around 15% of total prevalent TB. In SA communities, where background TB prevalence was higher (1.53%), the proportional contribution was smaller (5.6%) despite a similar absolute excess TB prevalence among PLHIV on ART. These contrasts highlight that the public health implications of excess TB prevalence among PLHIV on ART can vary by setting: in lower-TB-prevalence environments, even modest excess absolute TB prevalence in PLHIV on ART may represent a substantial fraction of the total TB prevalence, whereas in higher-TB-prevalence environments, the impact of a modest excess absolute TB prevalence may be less pronounced at the population level. This has implications for targeting TB prevention and screening strategies, suggesting that context-specific approaches are required.

Sub-group analyses showed broadly similar patterns of excess TB prevalence across demographic strata; including among males and females, older and younger individuals, and in Zambian and SA communities. When stratified by survey timelines, the estimated PD was 0.73% (95% CI: 0.27, 1.20) prior to the COVID-19 pandemic and 0.32% (95% CI: −0.14, 0.79) during the COVID-19 pandemic. However, the confidence intervals overlapped substantially, and there was no statistical evidence of heterogeneity in excess TB prevalence between the two periods. Nonetheless, pandemic-related disruptions to TB and HIV services, changes in healthcare access, and altered patterns of healthcare-seeking may have influenced TB detection during this period [43–47].

This analysis had several strengths. First, using inverse probability of treatment weighting (IPTW) within a causal inference framework allowed robust estimation of absolute differences in TB prevalence, while reducing the reliance on correct model specification of the relationship between the outcome (prevalent TB) and explanatory variables, which is particularly valuable for rare outcomes such as prevalent TB [48]. Second, our propensity score model incorporated key interaction terms informed by prior knowledge of TB prevalence patterns in the study communities [16,20,49], and covariate balance diagnostics indicated successful balance across measured characteristics, reducing observable confounding and strengthening the plausibility of the estimates. Finally, a large sample size and relatively common exposure (15.2% of the study population) further improved precision and minimized concerns about positivity violations.

The study also had several limitations. *First*, the identification of individuals who were PLHIV and on ART was based on self-reported information. Evidence from these communities suggests that approximately 10% of self-reported ART users in these communities were non-adherent to ART and approximately 12% were not virally suppressed [50]. This potential misclassification of the treated group may have led to an underestimation of the protective effect of ART (if taken as intended). *Second*, defining ART use as a binary (yes/no) variable means that this group encompasses individuals with varying durations of treatment, adherence histories, and immune recovery. Thus, our ATT estimate reflects an average effect under routine program conditions, not the biological efficacy of fully suppressive ART. If many participants were recent initiators or had incomplete immune reconstitution, our estimates may overstate long-term risk under stable ART. This highlights that our findings represent real-world programmatic outcomes rather than ideal treatment effects. Future work incorporating longitudinal information on ART duration, historical viral load trajectories, and objective markers of immune status could further refine these overall estimates. *Third*, the cross-sectional design precluded definitive establishment of the temporal sequence between HIV acquisition, ART initiation, and development of prevalent TB. Because exposure and outcome were measured at the same survey visit, interpretation of the estimated contrasts relies on assumptions regarding temporal ordering that cannot be directly verified from these data. Consequently, the estimand represents a cross-sectional contrast in point-prevalence state occupancy. This distinction is important because prevalent TB reflects not only disease occurrence (i.e., disease incidence), but also the cumulative influence of health-system and programmatic factors that determine the duration of undetected disease within the community. In routine HIV care settings, point TB prevalence is shaped by factors such as retention in HIV care, the frequency and sensitivity of TB screening, TB diagnostic delays, and TB treatment initiation timelines. As such, the estimated TB prevalence contrasts quantify the residual burden of prevalent TB in the context of routine care at a particular point in time. This provides a complementary, programmatically relevant perspective on the prevailing reservoir of TB disease among PLHIV who are on ART that would not be fully captured by longitudinal TB incidence measures alone. *Fourth*, because the outcome was TB prevalence, the estimates reflect a combination of TB incidence, disease duration, diagnostic delays, and survival, introducing the potential for survivor selection bias. Our target population comprises survey participants who were alive and reported receiving ART at the time of measurement. Individuals who died prior to the survey are necessarily absent from this population, and if factors associated with survival are also associated with TB prevalence, the estimated contrast may differ from that which would have been observed in the broader population of people who acquired HIV and started ART. Although ART substantially improves survival, survivor selection or depletion of susceptible individuals cannot be ruled out. Consequently, our findings apply specifically to surviving survey participants living with HIV and receiving ART. *Fifth*, although our adjustment set was pre-specified using a causal DAG to control for key shared determinants of HIV status and TB, conditional exchangeability cannot be empirically verified. Residual confounding from unmeasured factors or measurement error in derived covariates like SES, crowding index, or alcohol use disorder, may therefore remain [51–53]. *Finally*, we assumed no interference – i.e., that one individual’s HIV status does not affect another’s TB risk. Given that TB is an infectious disease with airborne transmission, this assumption is likely violated. Thus, our estimates may not capture the indirect community-level effects of ART. Future work applying methods that account for interference (e.g., network-based or general equilibrium models) could better estimate the full public health impact of ART on TB prevalence [54].

In conclusion, these findings highlight the importance of selecting the estimand most appropriate for the research question being addressed - in this case, the ATT. Using this estimand, we demonstrated an absolute excess in the prevalence of pulmonary TB among PLHIV on ART compared to the counterfactual scenario in which those same individuals were HIV-negative. While this excess prevalence is modest in absolute terms at the individual level, it contributes considerably to overall TB prevalence in settings where a substantial proportion of the population is living with HIV and receiving ART. The findings underscore the need for targeted TB prevention, screening, and treatment strategies tailored to PLHIV, alongside broader community-wide TB control efforts.

## Supporting information

S1 STROBE Checklist STROBE, strengthening the reporting of observational studies in epidemiology. (DOC)

S1 Appendix Supplementary material for the study. Includes additional details on variable derivation, propensity-score specification and causal assumptions, covariate balance and positivity diagnostics, inverse probability weights, ATE and ATT analyses, and sensitivity analysis using E-values, together with supporting tables and figures.
(PDF)
