# Supplementary material for: Quantifying excess TB prevalence among people living with HIV on ART in the universal ART era: Evidence from a TB prevalence survey in Zambia and South Africa
Source: PLOS Glob Public Health. 2026 Sep 21;6(9):e0006833. doi: 10.1371/journal.pgph.0006833 (PMC13592586; doi:10.1371/journal.pgph.0006833)
Supplement: Supplementary file 1 [file pgph.0006833.s002.pdf]

Supplementary material for:

Quantifying excess TB prevalence among people living with HIV on ART in the universal ART era: Evidence from a TB prevalence survey in Zambia and South Africa.

Thomas Gachie, Eveline Klinkenberg, Kwame Shanaube, Linda Mureithi, Peter J. Dodd, Maria Ruperez, Ab Schaap, Richard Hayes, Helen Ayles, Jonathan Bartlett & Sian Floyd

Table of Contents

Text A: Socio-economic status (SES) 3
List of items used in the PCA 3
Zambia 4
1] Correlation analysis 4
Fig A: Heatmap of Pearson correlations of PCA items in Zambia 4
2] PCA 1 (Model 1) 5
Fig B: Summary results for a PCA of all variables 5
3] PCA 2 (Model 2) 5
Fig C: Summary results for PCA of all variables excluding water, building type and motorcycle 5
South Africa 6
1] Correlation analysis 6
Fig D: Heatmap of Pearson correlations of PCA items in South Africa 6
2] PCA 1 (Model 1) 6
Fig E: Summary results for a PCA of all variables 6
3] PCA 2 (Model 2) 7
Fig F: Summary results for PCA of all variables excluding water, building type and motorcycle 7
Text B: Alcohol Use Disorder 7
Fig G: Overall distribution of the Audit score 9
Table A: Alcohol use disorders identification test (AUDIT) score test. Overall and by country 9
Text C: Household crowding i.e. Crowding index 9
Fig H: Overall crowding index distribution 10
Text D: The propensity score model 10
Epidemiological and structural justification of the adjustment set 10
The backdoor criterion 10
Justification of the minimally sufficient adjustment set 11
Propensity score model specification 12
Causal assumptions. 12
Correct specification of PS model. 13

Causal estimator 13
Table B: Distribution of HIV positivity on ART and prevalent TB (observed and imputed) by baseline characteristics, with proportion of missing outcome data, among survey participants 14
Fig I: Covariate balance before weighting. 17
Fig J: Proportion of parameters with initial covariate imbalance, categorized by ASMD level 18
Fig K: Covariate balance after weighting 19
Table C: Logistic regression model fitted to estimate the overall propensity score 20
Text E: Assessment of positivity and inverse probability weights 21
Fig L: Propensity score overlap plot, overall 22
Fig M: Propensity score overlap plot by country 22
Fig N: Propensity score overlap plot by Sex 23
Fig O: Propensity score overlap plot by Age 23
Fig P: Propensity score overlap plot by COVID-19 period 24
Table D: Summary statistics for the inverse probability weights used in ATT and ATE estimands, overall and separately for each subgroup 25
Fig Q: Distribution of ATT weights by exposure group 26
Fig R: Distribution of ATE weights by exposure group 27
Text F: Comparison of ATE and ATT estimates 28
Fig S: ATE and ATT prevalence difference estimates comparing TB prevalence between PLHIV on ART and HIV-negative individuals: overall and by subgroup 29
Text G: Sensitivity analysis 29
E-values 29
Results 30
References 30

Text A: Socio-economic status (SES)

The household-level socio-economic status (SES) variable was derived using Principal Component Analysis (PCA) based on housing characteristics and asset ownership variables. Because patterns of asset ownership and housing quality differ between settings, PCA was conducted separately for Zambia and South Africa (SA) to allow the SES index to reflect relative wealth within each country.

The first principal component (PC1) was extracted and used as the SES score, capturing the largest amount of variance across households based on asset ownership and housing characteristics [1]. All categorical variables were first converted into binary (dummy) variables before inclusion in the PCA to ensure uniform scaling.

The following household characteristics and assets were included in the PCA:-

List of items used in the PCA

Question	Variable name	Binary variable
1. Which of the following best describes the main type of building that this household occupies?	Building type	1=single unit/brick (8), cluster/multi-unit (2), flat (3), servant quarters (6), workers' hostel (10)<br>0=traditional hut (9), shack (7), caravan/tent (1) other (5)
2. What is the main type of flooring for this household?	Flooring type	1=parquet (6), lino (3), cement (1), tile (7)<br>0=dirt/earth (2), wood/plank (8), other (5)
3. What is the main source of drinking water for this household?	Drinking source	1=Piped indoors (6) Standpipe/tap within plot (8) Communal tap (4) Borehole (2) Protected well (7)<br>0=Unprotected/shallow well/dam/lake/pan (9) Bowser/Tanker (3) Other (1)
4. What is the main source of energy used for cooking?	Energy source	1=2 Electricity (mains) (3) Electricity (individual solar) (2) Gas (4)<br>0= Paraffin (8) Charcoal (1) Wood (9) Other (7) No cooking done in household (6)
5. What is the main toilet facility used in your household?	Toilet type	1=Own flush toilet (6) Shared flush toilet (9) own VIP latrine (5) Shared VIP latrine (10)<br>0=Own pit latrine (7) Shared pit latrine (11) Pail/bucket (8) Communal chemical latrine (2) Bush (1)
6. Does any member of your household have access to the following items in good working order:	Asset ownership	
	Working Cell phone	1 = Yes<br>0 = No
	Bicycle	1 = Yes<br>0 = No
	Motorcycle or scooter	1 = Yes<br>0 = No
	Car/bakkie in SA	1 = Yes<br>0 = No
	Electricity to house	1 = Yes<br>0 = No
	TV	1 = Yes<br>0 = No

	Fridge/Freezer	1 = Yes<br>0 = No
	Radio	1 = Yes<br>0 = No
	Computer/laptop	1 = Yes<br>0 = No
	CD or MP3 player	1 = Yes<br>0 = No
	Stereo/Cassette/Other music player	1 = Yes<br>0 = No
	Working Internet	1 = Yes<br>0 = No

The same list of household items was used for PCA calculation for Zambia and SA, however, separate PCA models were fitted.

As an initial step, correlation analyses were performed to examine relationships between candidate PCA variables. Pearson correlation heatmaps were generated for Zambia and SA (Figs A and D) to assess the extent to which asset and housing indicators captured shared underlying variation. Moderate (i.e. pearson correlation <0.8) were observed among several variables in both countries, particularly among indicators of housing quality (e.g., building type, flooring, water source, sanitation) and ownership of household goods (e.g., electricity, television, refrigerator, and computer).

Zambia

1] Correlation analysis

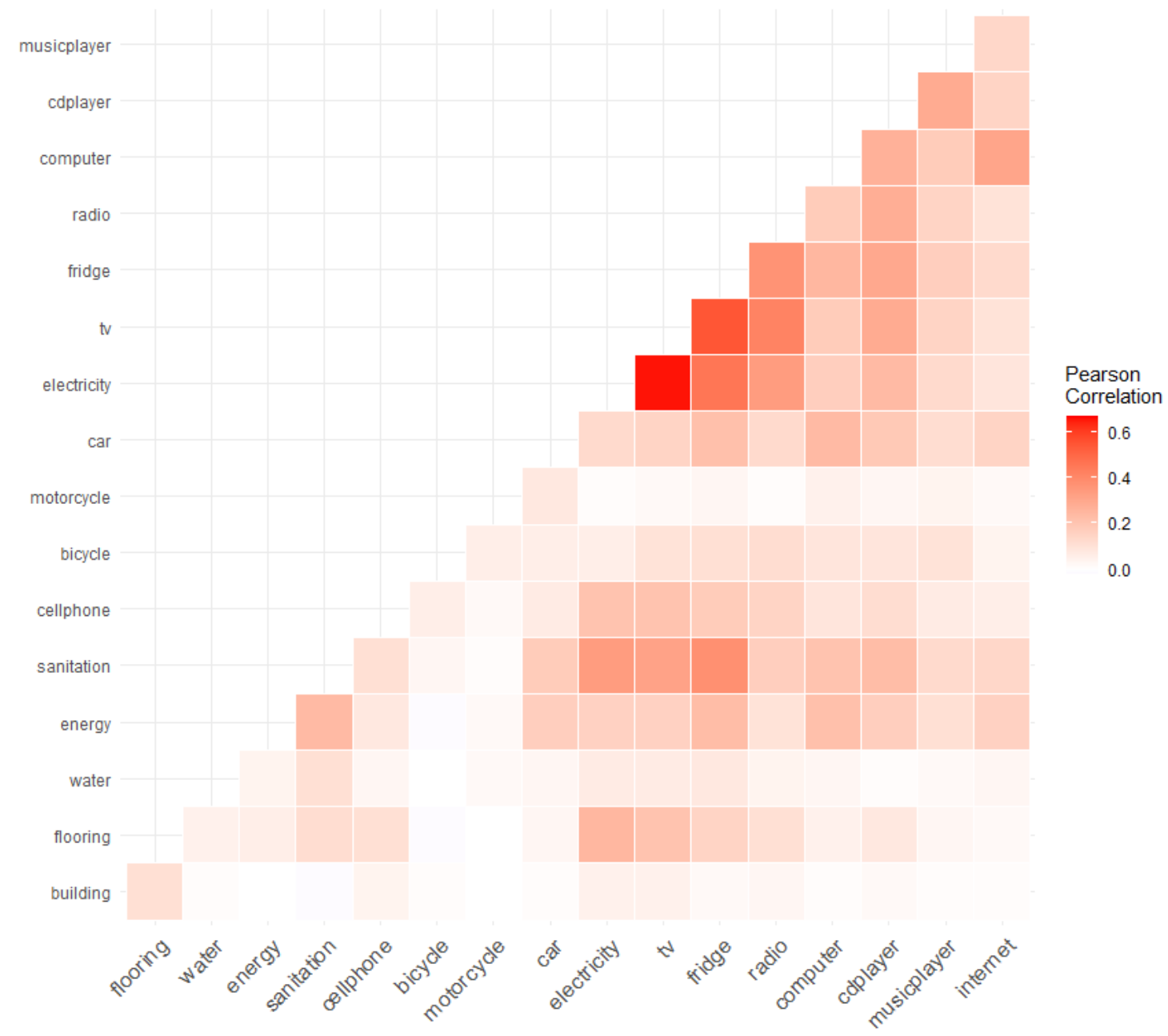

Fig A: Heatmap of Pearson correlations of PCA items in Zambia

2] PCA 1 (Model 1)

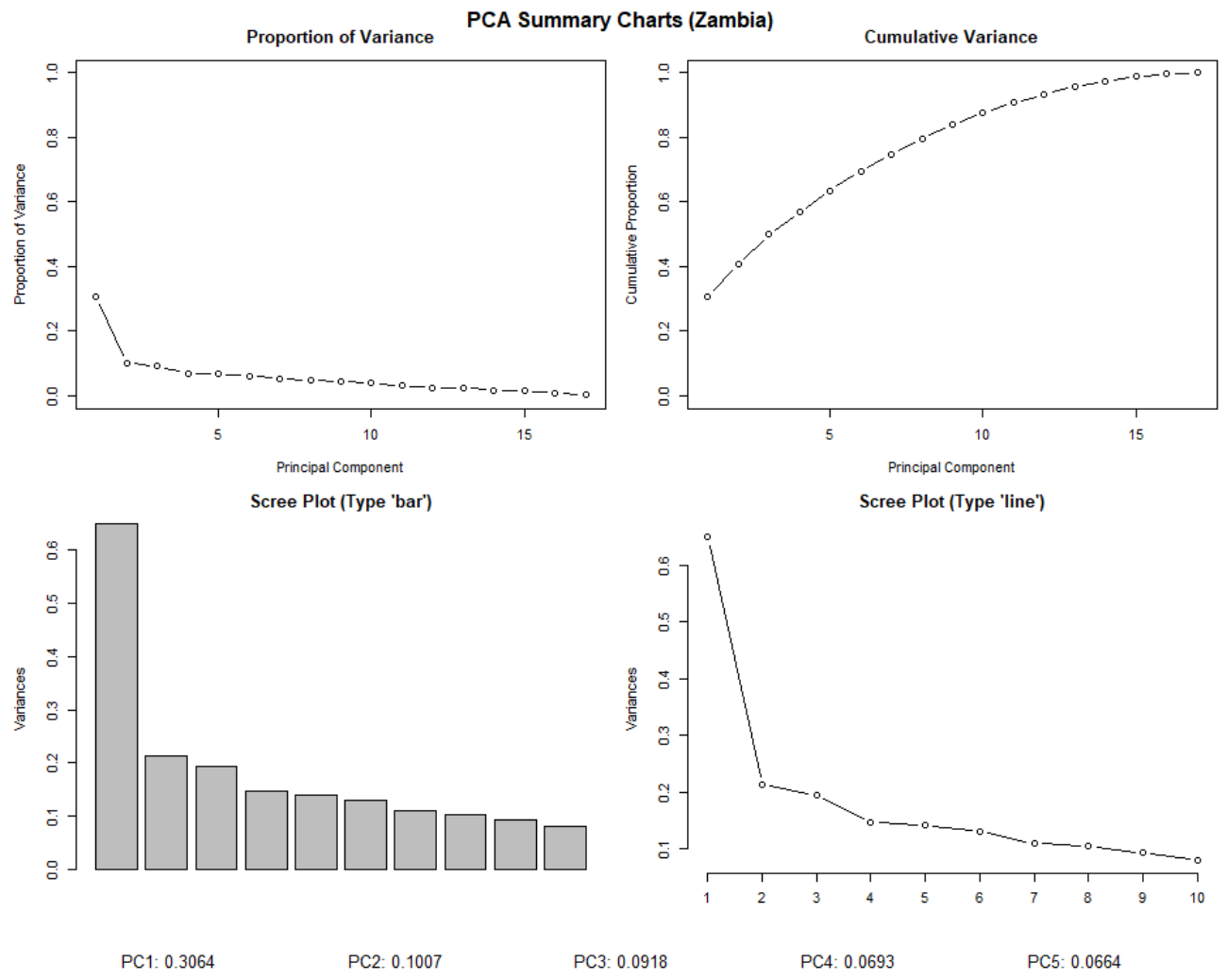

Fig B: Summary results for a PCA of all variables

3] PCA 2 (Model 2)

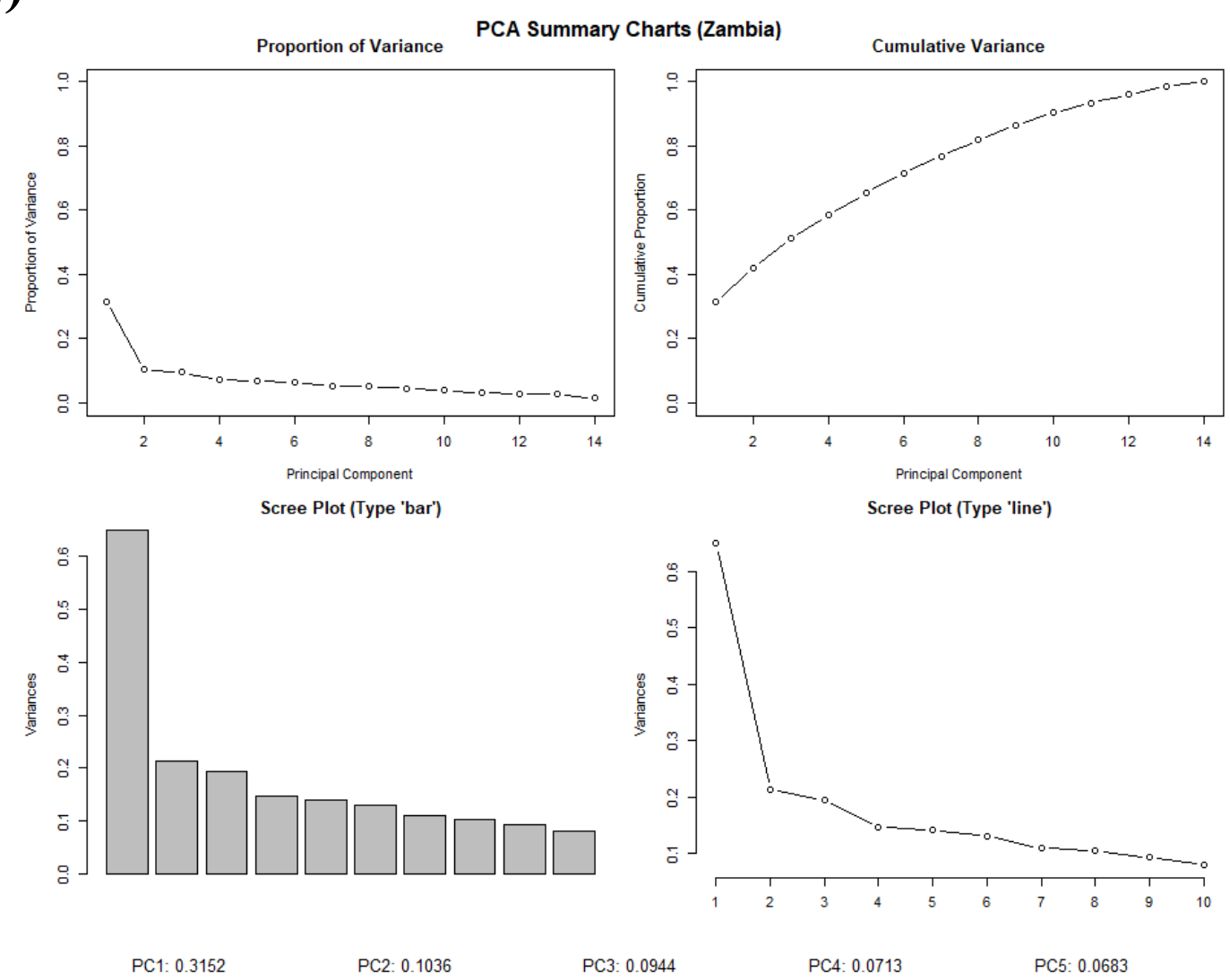

Fig C: Summary results for PCA of all variables excluding water, building type and motorcycle

South Africa

1] Correlation analysis

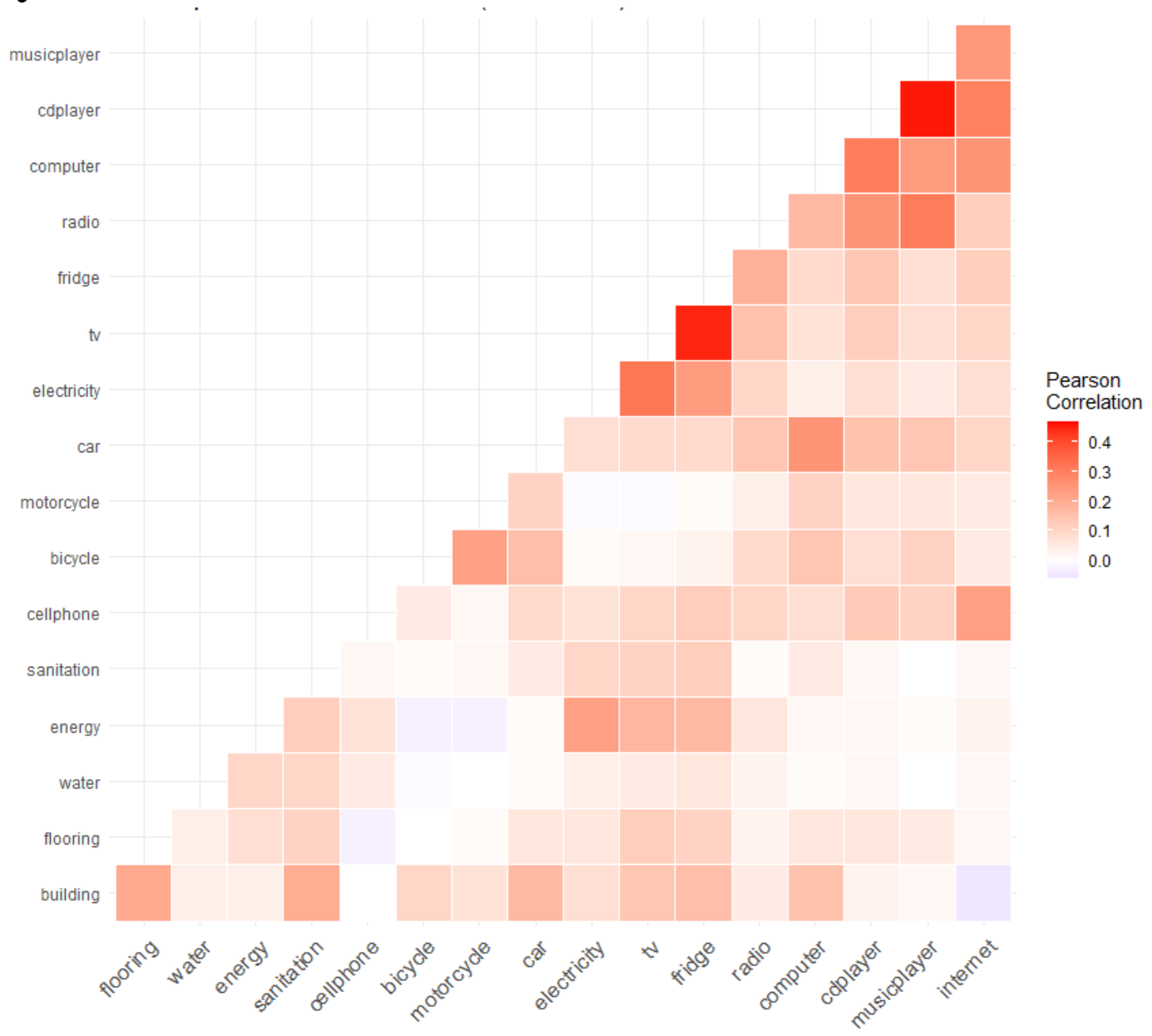

Fig D: Heatmap of Pearson correlations of PCA items in South Africa

2] PCA 1 (Model 1)

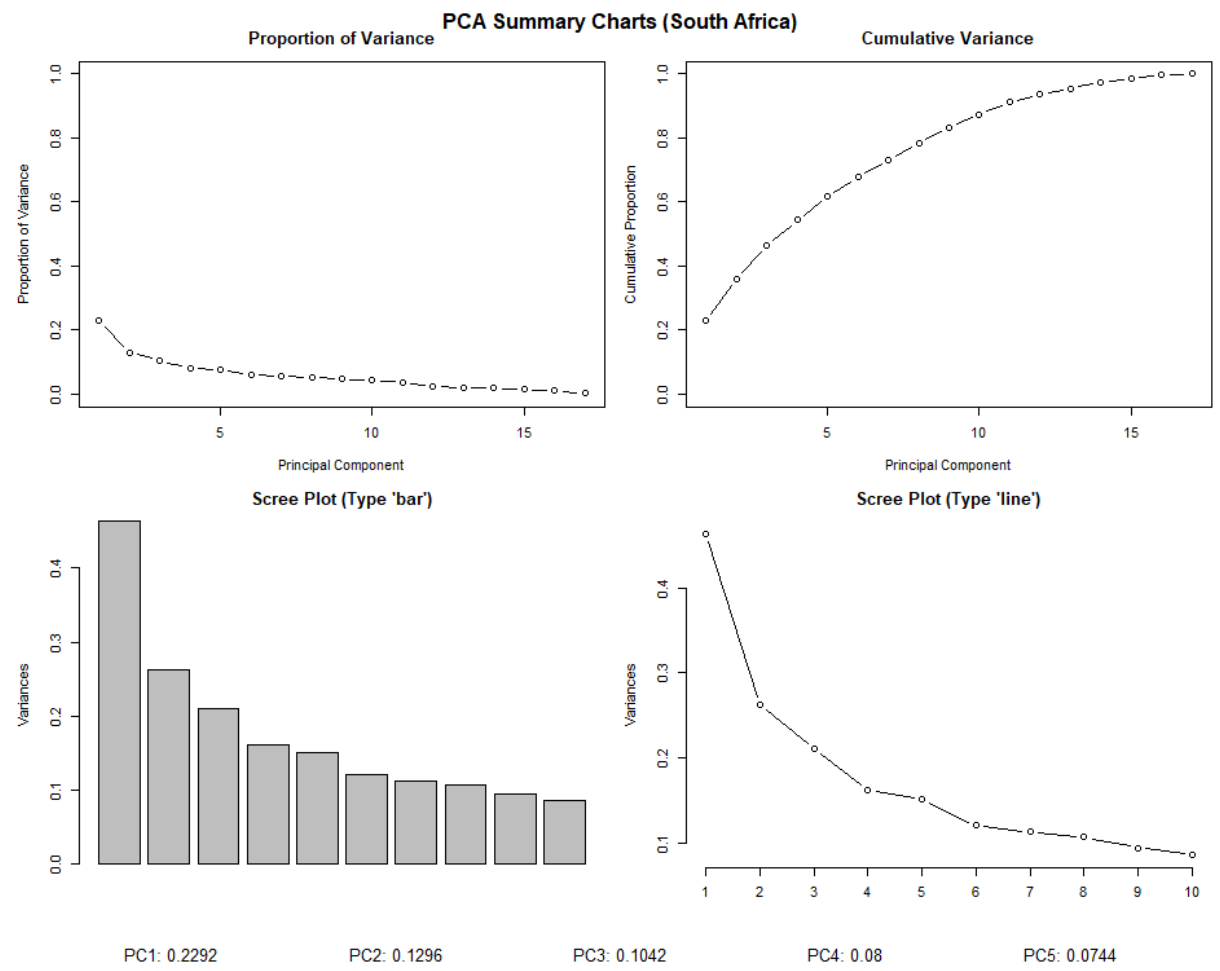

Fig E: Summary results for a PCA of all variables

3] PCA 2 (Model 2)

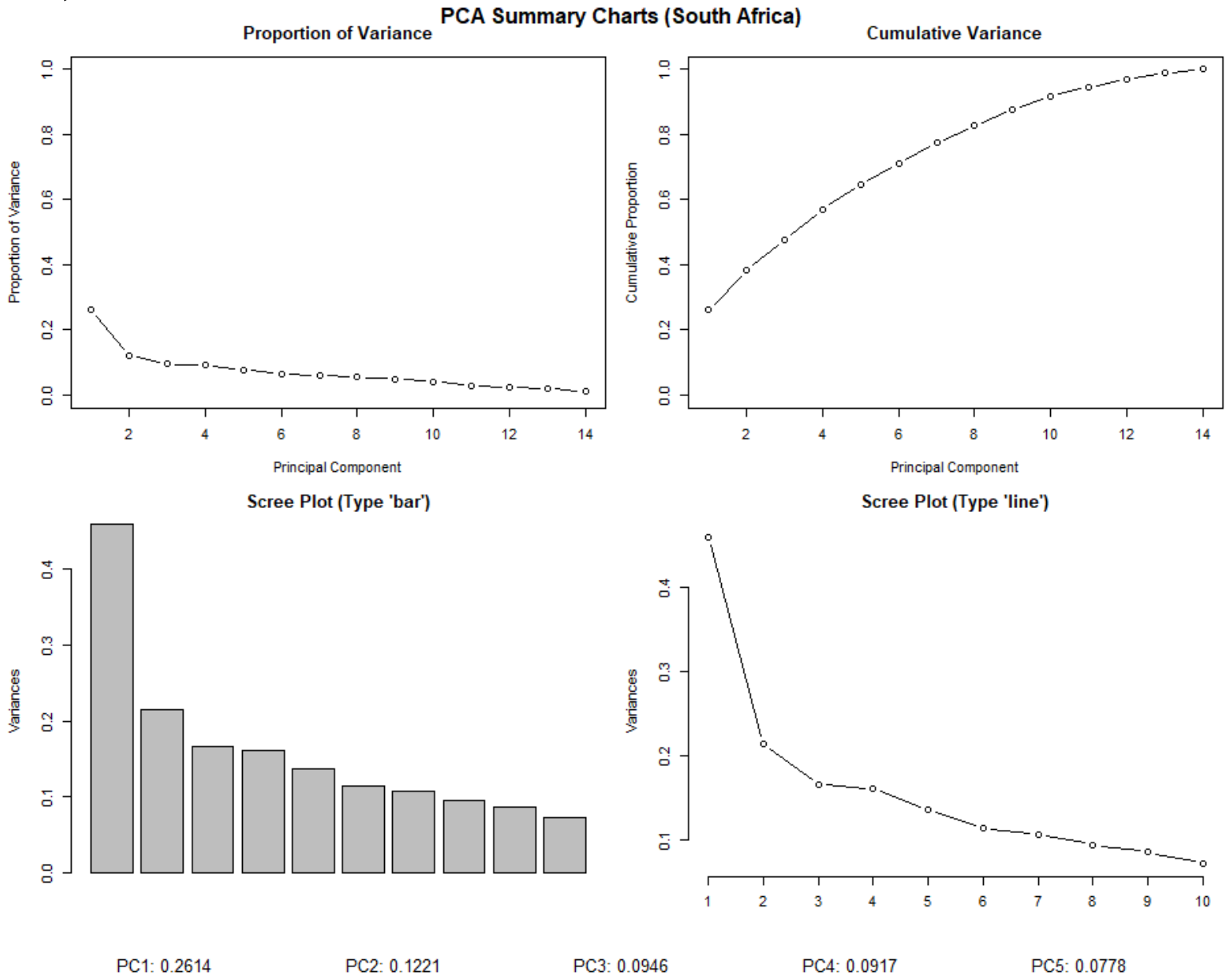

Fig F: Summary results for PCA of all variables excluding water, building type and motorcycle

Two PCA models were then explored in each country. The first model (PCA 1) included all candidate variables. A second model (PCA 2) excluded selected variables (water source, building type, and motorcycle ownership) that showed weaker or inconsistent correlations with the remaining indicators. The proportion of variance explained by the first principal component (PC1) in Zambia increased marginally from 30.6% to 31.5% while that of SA increased from 22.9% to 26.1%.

In Zambia, the PCA model including all variables (PCA 1) was retained, as it adequately captured variation in household assets and housing conditions. In SA, the reduced model excluding water source, building type, and motorcycle ownership (PCA 2) was selected, as these variables contributed less consistently to the shared wealth construct and their removal improved the proportion of variance explained.

Text B: Alcohol Use Disorder

Alcohol use disorder was defined using the alcohol use disorders identification test (AUDIT) [2,3]

Below are the questions used in the survey: -

**Question**	**Response**	**score**
1] How often do you have a drink containing alcohol?	Never<br>Monthly or less<br>2 to 4 times a month<br>2 to 3 times a week<br>4 or more times a week	0 points<br>1 point<br>2 points<br>3 points<br>4 points
2] How many drinks containing alcohol do you have on a typical day when you are drinking?	1 or 2<br>3 or 4<br>5 or 6<br>7 to 9<br>10 or more	0 points<br>1 point<br>2 points<br>3 points<br>4 points

3] How often do you have six or more drinks on one occasion?	Never<br>Less than monthly<br>Monthly<br>Weekly<br>Daily or almost daily	0 points<br>1 point<br>2 points<br>3 points<br>4 points
4] How often during the last 12 months have you found that you were not able to stop drinking once you had started?	Never<br>Less than monthly<br>Monthly<br>Weekly<br>Daily or almost daily	0 points<br>1 point<br>2 points<br>3 points<br>4 points
5] How often during the last 12 months have you failed to do what was normally expected from you because of drinking?	Never<br>Less than monthly<br>Monthly<br>Weekly<br>Daily or almost daily	0 points<br>1 point<br>2 points<br>3 points<br>4 points
6] How often during the last 12 months have you needed a first drink in the morning to get yourself going after a heavy drinking session?	Never<br>Less than monthly<br>Monthly<br>Weekly<br>Daily or almost daily	0 points<br>1 point<br>2 points<br>3 points<br>4 points
7] How often during the last 12 months have you had a feeling of guilt or remorse after drinking?	Never<br>Less than monthly<br>Monthly<br>Weekly<br>Daily or almost daily	0 points<br>1 point<br>2 points<br>3 points<br>4 points
8] How often during the last 12 months have you been unable to remember what happened the night before because you had been drinking?	Never<br>Less than monthly<br>Monthly<br>Weekly<br>Daily or almost daily	0 points<br>1 point<br>2 points<br>3 points<br>4 points
9] Have you or someone else been injured as a result of your drinking?	No<br>Yes, but not in the last year<br>Yes, during the last year	0 points<br>2 points<br>4 points
10] Has a relative or friend or a doctor or another health worker been concerned about your drinking or suggested you cut down?	No<br>Yes, but not in the last year<br>Yes, during the last year	0 points<br>2 points<br>4 points

The sum of the score for the 10 questions was calculated for each participant, and then categorized as:-

- Low risk (0 to 7 points): “You probably do not have a problem with alcohol. Continue drinking in moderation or not at all”.
- Medium risk (8 to 15 points): “You may drink too much on occasion. This may put you or others at risk. Try to cut down on alcohol or stop drinking completely”.
- High risk (16 to 19 points): “Your drinking could lead to harm, if it has not already. It is important that you cut down on alcohol or stop drinking completely. Ask your doctor or nurse for advice on how best to cut down”.
- Addiction likely (20+ points): “It is likely that your drinking is causing harm. Speak to your doctor or nurse or an addiction specialist. Ask about medications and counselling that can help you stop drinking. If you are dependent on alcohol, do not stop drinking without the help of a health care professional”.

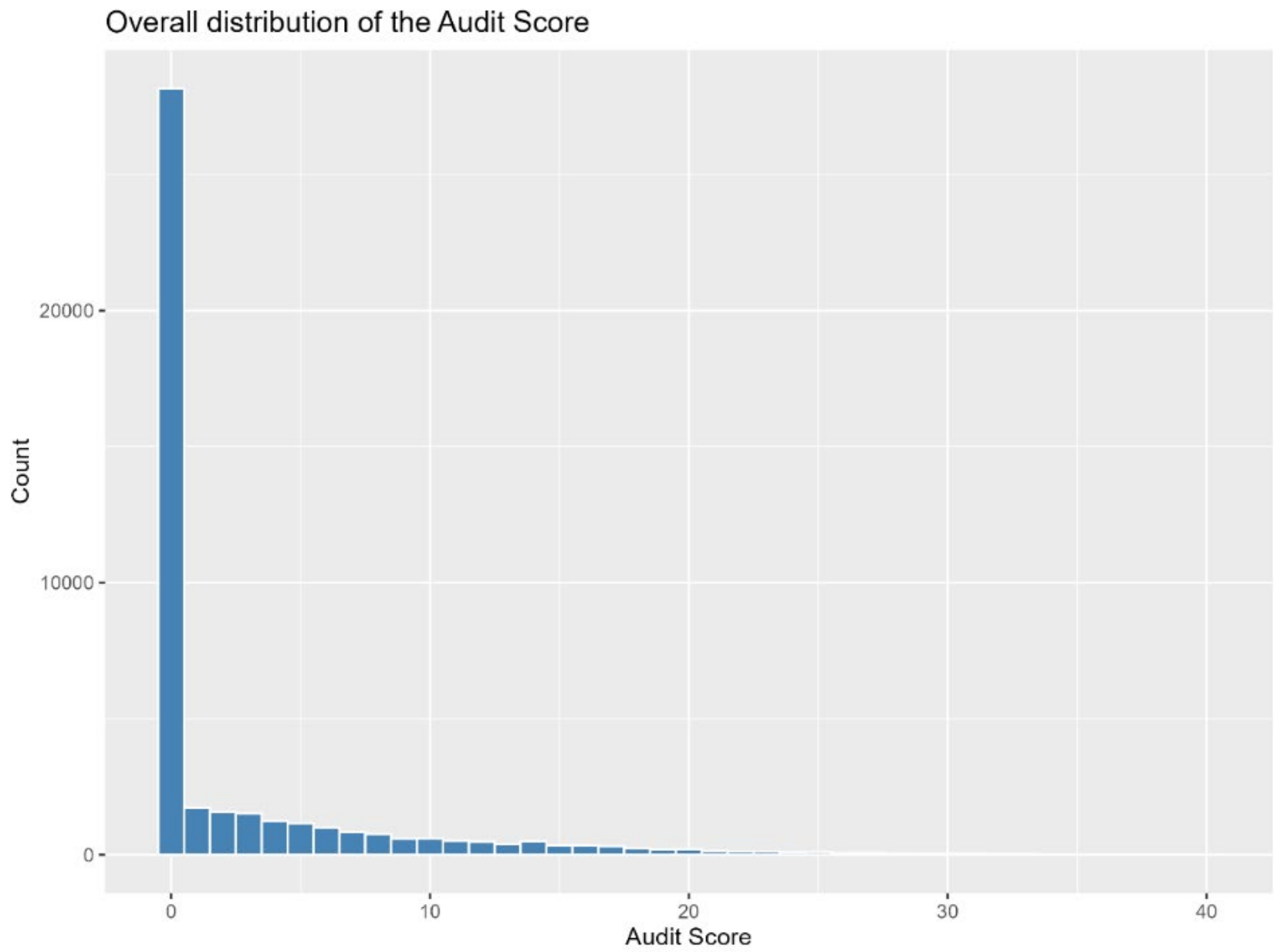

Fig G: Overall distribution of the Audit score

Table A: Alcohol use disorders identification test (AUDIT) score test. Overall and by country

	**Overall**		**Zambia**		**South Africa**	
**AUDIT score**	**n**	**%**	**n**	**%**	**n**	**%**
Low risk:0-7	37,204	85.3%	24,713	88.8%	12,491	79.1%
Medium risk:8-15	4,186	9.6%	1,911	6.9%	2,275	14.4%
High risk:16-19	1,080	2.5%	486	1.8%	594	3.8%
Addiction likely:20-40	1,147	2.6%	717	2.6%	430	2.7%

Note: % = column percentages; n = frequency counts

Text C: Household crowding i.e. Crowding index

The *crowding index*, a household-level variable derived from the household questionnaire, was calculated by dividing the number of people living in the household (enumerated individuals) by the number of sleeping rooms.

Below are distributions of crowding index, overall, by country, sex, age and COVID-19 period

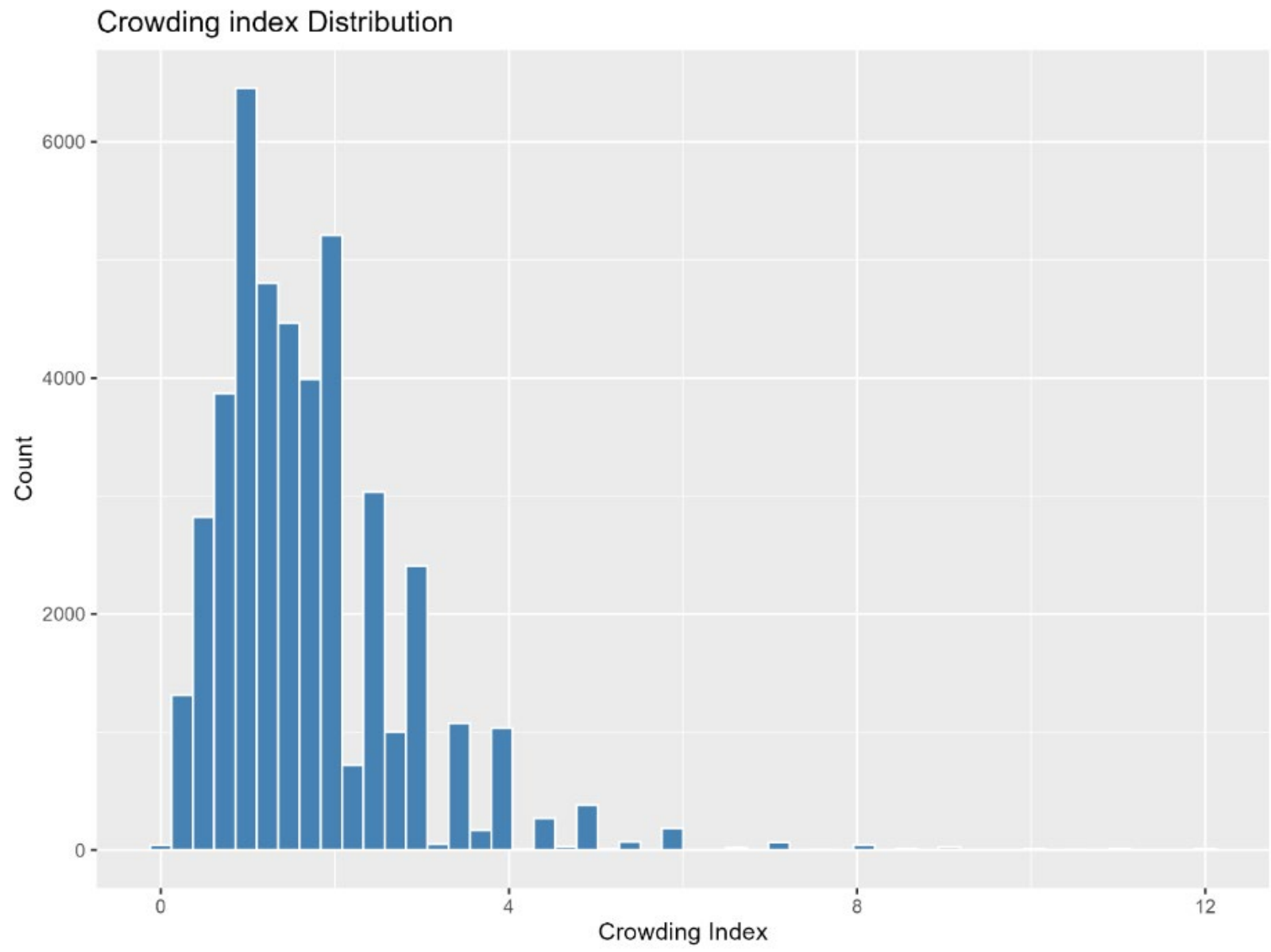

Fig H: Overall crowding index distribution

Text D: The propensity score model

Epidemiological and structural justification of the adjustment set

The backdoor criterion

To isolate the effect of our composite exposure on prevalent TB, we applied Judea Pearl's back-door criterion to our DAG (Fig 1, main text).

In causal inference, a "back-door path" is any non-causal, associative pathway that starts with an arrow pointing into the exposure and winds its way to the outcome (E.g. exposure ← confounder → outcome). Information flowing backward through these paths introduces confounding bias, creating a spurious statistical association.

The back-door criterion provides a formal mathematical test to determine whether a specific set of variables is sufficient to block or "close" all such confounding pathways. A set of variables X satisfies the back-door criterion relative to an exposure A and an outcome Y if it meets two strict conditions:

- No descendants of the exposure: No variable in X can be a descendant of A. Adjusting for variables that occur after or are caused by the exposure (mediators) would accidentally block the real causal pathways we aim to capture
- Block all back-door paths: The variables in Xmust block every open path between A and Y that contains an arrow pointing into A.

In a DAG, a path is blocked either by conditioning on a non-collider or by leaving an unconditional collider alone (i.e. exposure → collider ← outcome), as colliders naturally block information flow unless stratified upon. By identifying the minimally sufficient adjustment set that meets these conditions, we close all back-door paths, mathematically forcing any remaining statistical correlation to reflect the true, unconfounded effect – specifically the ATT.

Justification of the minimally sufficient adjustment set

Potential confounding variables were identified using a directed acyclic graph (DAG) informed by existing epidemiological evidence and subject-matter knowledge (Fig 1, main text). The DAG was used to identify a minimally sufficient adjustment set of variables that could plausibly influence both the exposure definition (living with HIV and receiving ART versus HIV-negative) and prevalent TB. These variables were age, sex, country and community of residence, socio-economic status (SES), education, occupation, marital status, alcohol use disorder, smoking status, and household crowding index.

The rationale for including each variable is outlined below.Age and sex, these variables function as foundational biological and epidemiological determinants of both HIV transmission dynamics (including subsequent engagement and long-term retention in ART infrastructure) and the biological risk of developing active pulmonary TB. Adult men in low- and middle-income countries (LMICs) experience more than twice the point prevalence of TB compared to women [4,5], Concurrently, older age is strongly associated with superior long-term ART adherence [6], whereas male sex is an empirical driver of non-adherence and clinical attrition [7].

Country of residence (i.e. Zambia vs. South Africa) acts as a macro-level structural confounder that directly influences exposure state occupancy via distinct national healthcare policies, varying timelines of universal test-and-treat (UTT) implementation, and clinical capacity. Simultaneously, it serves as a proxy for broad, country-level geographic variations in background TB transmission dynamics and baseline community prevalence. On the other hand, community of residence, serves as a vital micro-level spatial confounder nested within country. Localized community factors - including neighbourhood-level HIV prevalence, localized forces of metabolic or nutritional vulnerability, and physical proximity to health facilities - shape an individual's likelihood of successfully accessing and remaining on ART, while concurrently dictating the local environmental force of *Mycobacterium tuberculosis* infection [4,8].

Household wealth index as a proxy of socioeconomic position, highest level of education, and occupation variables, are modelled as distinct structural confounders that shape a participant's regular interaction with the healthcare system, impacting the likelihood of HIV diagnosis (if positive) and retention on ART. Lower SES has been shown to be associated with high rates of TB [4,9,10] and lower levels of ART adherence [11]. Concurrently, all these three variables dictate living and working conditions - such as overcrowded environments or poorly ventilated occupational spaces - which directly alter the risk of exposure to MTB and subsequent progression to active TB disease. By adjusting for these variables, we effectively block backdoor pathways driven by differential healthcare access and socio-economic vulnerability.

Marital status is a social determinant that influences stable engagement and longitudinal retention within clinical HIV care pipelines while reflecting shared household-level social support networks and exposure dynamics. Committed or cohabiting relationships are associated with distinct probabilities of HIV status awareness [12], and significantly higher levels of long-term ART adherence[13], while simultaneously acting as a proxy for shared household-level risk factors associated with close-contact TB exposure [14]

Alcohol use disorder is a chronic behavioural confounder that heavily impacts clinical adherence, health-seeking behaviour, and regular retention within routine HIV care programs [7]. Simultaneously, chronic alcohol misuse acts as a potent biological driver of TB progression by suppressing cell-mediated immunity, directly accelerating the transition from latent infection to active, prevalent pulmonary disease [10].

Smoking is a well-established risk factor for active TB through its adverse effects on pulmonary and

immune function [10]. We did not hypothesize a direct causal relationship between smoking and the exposure state (living with HIV and receiving ART versus HIV-negative), and therefore no direct arrow from smoking to the exposure node was included in the DAG. However, smoking was unevenly distributed between the exposure groups in the observed data (Table 1, main text), likely reflecting broader socio-demographic and behavioural differences captured elsewhere in the causal structure. Given its strong association with prevalent TB, smoking was included in the propensity score model to improve covariate balance and reduce the potential for residual confounding.

Finally, crowding index is a baseline environmental variable captures indoor residential density, serving as a profound direct driver of the airborne transmission dynamics, proximity, and physical exposure risks required to contract MTB and develop prevalent TB within the household [10]. To ensure a valid adjustment set and avoid overadjustment bias, crowding is modelled alongside SES as a co-dependent baseline structural characteristic rather than a downstream mediator, thereby properly isolating the independent environmental pathway of airborne exposure from broader socioeconomic health-system access.

Propensity score model specification

The propensity score (PS) was estimated using a logistic regression model. The PS represents the conditional probability of being an individual living with HIV and on ART given the observed potential confounders. When estimating the PS from the observed data, the following recommendations were considered:

(i) Include at least all baseline covariates that could confound the relationship between the exposure and outcome
(ii) Include covariates known to be associated with the outcome only, to improve precision (e.g. the inclusion of crowding index).
(iii) Avoid including covariates associated with the exposure only as they lead to an unnecessary increase in bias and variance of the exposure effect estimate
(iv) Include any interactions or non-linear functional forms of the covariates
(v) Finally, avoid relying on the variable selection methods that rely on the p-value as they may lead to important variables being dropped, but to rely on existing literature and expert knowledge on the topic. The use of a DAG is therefore recommended. Rubin refers to this as the design phase as it does not involve modelling the outcome [15–17].

In this analysis, a logistic regression model was fitted with HIV status (PLHIV on ART vs. HIV-negative) as the outcome. The following independent variables were included to control for potential confounding: age, sex, country and community of residence, marital status, education, occupation, alcohol use disorder (AUD), and socioeconomic status (SES). Smoking and household crowding were also included in the PS model, despite being primarily associated with TB status (potentially to improve precision of the estimates). We also included the following interaction terms: age and sex, sex and country, and age and country.

Causal assumptions.

For valid estimation of the Average Treatment Effect on the Treated (ATT) (and Average Treatment Effect (ATE)), two key assumptions are necessary.

Rosenbaum and Rubin have shown that under Stable unit treatment value assumption (SUTVA) and the strong ignorability assumption, if correctly specified, the PS is the coarsest balancing score such that, for a given PS, the distribution of baseline covariates is the same regardless of the exposure (14).

1. **Strong ignorability (which includes both conditional exchangeability and positivity assumptions)**

Conditional exchangeability (or no unmeasured confounding)
Under this assumption, after conditioning on the observed covariates included in the PS model, the exposed and unexposed groups are assumed to be exchangeable in terms of their potential outcomes, meaning that if the unexposed had been exposed (or vice versa), their outcome (TB status) would have been similar to that of the actually exposed (or unexposed).

We can however not rule out unmeasured confounding, for instance, other unmeasured potential confounders that were not observed may include substance misuse, malnutrition and comorbidities like diabetes. We have however used the E-value framework for sensitivity analysis, to assess the robustness of our findings to potential unmeasured confounding (See findings in Text G below).

Positivity assumption (Overlap)
For every combination of covariates included in the PS model, there is a non-zero probability of an individual living with HIV and on ART or HIV-negative. This ensures that comparisons between exposure groups are possible across all strata of the population. PS overlap plots and covariate balance plots and statistics (i.e. Standardised mean difference) are used to ensure that this assumption is not violated.

2. **Stable unit treatment value assumption (SUTVA) (which includes both the no interference and consistency assumptions)**
   It is assumed that one individual's HIV status does not influence another individual's TB outcome (***no interference***), and that HIV status with ART is a well-defined, ***consistent*** definition of each level of the exposure, across all individuals. HIV negative status is defined after HIV diagnostic test, as negative, while PLHIV on ART is a self-reported variable.

   As detailed in the discussion section, being currently on ART is a self-reported variable and is subject to biases, where individuals who self-reported that they were currently on ART, may in fact have not been on ART.

Correct specification of PS model.

Both overlap and covariate balance plots are crucial diagnostic tools, not only for assessing the assumptions of exchangeability and overlap, but also providing insights into the adequacy and potential misspecification of the PS model itself.

As shown in the results section, the overlap and covariate balance plots, with absolute standardised mean differences (ASMD) of <10% suggest that the model was correctly specified [18]. ASMD is the absolute value of the standardised mean difference (SMD) which was calculated as:

$$ASMD = \frac{|\bar{X}_{HIV+} - \bar{X}_{HIV-}|}{\sqrt{\frac{S^2_{HIV+} - S^2_{HIV-}}{2}}}$$

Where $\bar{X}$ and S^2 denote the sample mean and variance for continuous variables (or prevalence for categorical variables) in each group. For categorical variables, variance was defined as $p(1-p)$, where p is the proportion of the exposure in that category.

Causal estimator

The estimated PS was used in the IPTW estimator to estimate the ATE and ATT as follows.

The estimate of the ATE was.

$$= \frac{1}{n}\sum_{i=1}^{n}\frac{A_iY_i}{PS_i} - \frac{1}{n}\sum_{i=1}^{n}\frac{(1-A_i)Y_i}{1-PS_i}$$

While estimate of the ATT was.

$$= \frac{1}{n}\sum_{i=1}^{n}A_iY_i - \frac{1}{n}\sum_{i=1}^{n}\frac{(1-A_i)PS_i \cdot Y_i}{1-PS_i}$$

Where, for each participant ($i = 1,2,\ldots,43{,}617$), Y_i denotes the outcome (i.e. TB status), A_i is an indicator (0/1) of the exposure (i.e. $A_i = 1$ if HIV status = PLHIV on ART, $A_i = 0$ if HIV status = HIV-negative), and PS_i is the estimated PS from the logistic regression model described above.

Below are the results of the PS model fitted, overall and separately for each sub-group.

Table B: Distribution of HIV positivity on ART and prevalent TB (observed and imputed) by baseline characteristics, with proportion of missing outcome data, among survey participants

Potential confounders		Prevalence of the exposure	Prevalence of the outcome		
	Overall	PLHIV, on ART	Proportion missing outcome	Prevalent TB (Observed)	Estimated Prevalent TB (Imputed)
	N	n/N (%)	n/N (%)	n/N (%)	n/N (%)
	**43,617**	**6,612 (15.2%)**	**640 (1.5%)**	**302 (0.7%)**	**381.1 (0.9%)**
**Country**					
Zambia	27,827	4,197 (15.1%)	251 (0.9%)	117 (0.4%)	140 (0.5%)
South Africa	15,790	2,415 (15.3%)	389 (2.5%)	185 (1.2%)	241.1 (1.5%)
**Community of residence**					
community 1	1,735	219 (12.6%)	20 (1.2%)	11 (0.6%)	12.8 (0.7%)
community 2	1,831	252 (13.8%)	19 (1%)	9 (0.5%)	9.9 (0.5%)
community 3	3,180	438 (13.8%)	22 (0.7%)	9 (0.3%)	10.2 (0.3%)
community 4	3,219	546 (17%)	63 (2%)	15 (0.5%)	19 (0.6%)
community 5	1,900	246 (12.9%)	30 (1.6%)	13 (0.7%)	15.1 (0.8%)
community 6	1,927	289 (15%)	22 (1.1%)	3 (0.2%)	3.5 (0.2%)
community 7	3,428	479 (14%)	18 (0.5%)	14 (0.4%)	17.7 (0.5%)
community 8	2,692	370 (13.7%)	20 (0.7%)	14 (0.5%)	18.4 (0.7%)
community 9	1,972	306 (15.5%)	12 (0.6%)	14 (0.7%)	16.1 (0.8%)
community 10	1,557	377 (24.2%)	10 (0.6%)	9 (0.6%)	10.9 (0.7%)
community 11	1,485	242 (16.3%)	3 (0.2%)	2 (0.1%)	2 (0.1%)
community 12	2,901	433 (14.9%)	12 (0.4%)	4 (0.1%)	4.3 (0.1%)
community 13	1,637	291 (17.8%)	19 (1.2%)	13 (0.8%)	18.6 (1.1%)
community 14	1,920	352 (18.3%)	35 (1.8%)	16 (0.8%)	18 (0.9%)
community 15	3,572	736 (20.6%)	65 (1.8%)	37 (1%)	48.9 (1.4%)
community 16	1,613	318 (19.7%)	64 (4%)	10 (0.6%)	14.5 (0.9%)

community 17	2,461	501 (20.4%)	55 (2.2%)	35 (1.4%)	49 (2%)
community 18	589	78 (13.2%)	25 (4.2%)	7 (1.2%)	12.1 (2.1%)
community 19	1,137	50 (4.4%)	36 (3.2%)	18 (1.6%)	20.6 (1.8%)
community 20	1,054	51 (4.8%)	40 (3.8%)	13 (1.2%)	16.2 (1.5%)
community 21	1,807	38 (2.1%)	50 (2.8%)	36 (2%)	43 (2.4%)
**Age Category**					
15-19	8,536	154 (1.8%)	51 (0.6%)	18 (0.2%)	26.3 (0.3%)
20-24	7,746	306 (4%)	61 (0.8%)	37 (0.5%)	48.0 (0.6%)
25-29	6,445	743 (11.5%)	77 (1.2%)	41 (0.6%)	51.6 (0.8%)
30-39	9,148	2,330 (25.5%)	137 (1.5%)	79 (0.9%)	100.2 (1.1%)
40-49	5,558	1,976 (35.6%)	113 (2%)	70 (1.3%)	85.2 (1.5%)
50-59	3,353	803 (23.9%)	90 (2.7%)	39 (1.2%)	47.2 (1.4%)
60+	2,831	300 (10.6%)	111 (3.9%)	18 (0.6%)	22.6 (0.8%)
**Sex**					
Male	16,876	1541 (9.1%)	288 (1.7%)	206 (1.2%)	248.8 (1.5%)
Female	26,741	5071 (19%)	352 (1.3%)	96 (0.4%)	132.3 (0.5%)
**Highest Level of education completed**					
None	1,332	222 (16.7%)	32 (2.4%)	17 (1.3%)	18.5 (1.4%)
Primary	9,546	1,744 (18.3%)	169 (1.8%)	69 (0.7%)	85.7 (0.9%)
Secondary	30,666	4,423 (14.4%)	428 (1.4%)	206 (0.7%)	265.5 (0.9%)
Higher	2,073	223 (10.8%)	11 (0.5%)	10 (0.5%)	11.5 (0.6%)
**Socio-economic Status**‡					
Lowest	8,645	1,486 (17.2%)	158 (1.8%)	72 (0.8%)	90.6 (1%)
Second	9144	1,451 (15.9%)	138 (1.5%)	79 (0.9%)	97.5 (1.1%)
Middle	8,753	1,296 (14.8%)	99 (1.1%)	49 (0.6%)	61.7 (0.7%)
Fourth	7,787	1,105 (14.2%)	120 (1.5%)	41 (0.5%)	56.0 (0.7%)
Highest	9,288	1,274 (13.7%)	125 (1.3%)	61 (0.7%)	75.2 (0.8%)
**Marital Status**					
Never married	24,451	2,527 (10.3%)	339 (1.4%)	197 (0.8%)	250.3 (1%)
Currently married or living as married	14,655	2,732 (18.6%)	214 (1.5%)	62 (0.4%)	81.7 (0.6%)
Divorced or separated	2,491	712 (28.6%)	36 (1.4%)	31 (1.2%)	35 (1.4%)
Widowed	2,020	641 (31.7%)	51 (2.5%)	12 (0.6%)	14.1 (0.7%)
**Alcohol use disorder**§					
Low risk:0-7	37,204	5,366 (14.4%)	490 (1.3%)	221 (0.6%)	278.5 (0.7%)
Medium risk:8-15	4,186	801 (19.1%)	99 (2.4%)	49 (1.2%)	63.9 (1.5%)
High risk:16-19	1,080	202 (18.7%)	24 (2.2%)	14 (1.3%)	17.5 (1.6%)
Addiction likely:20-40	1,147	243 (21.2%)	27 (2.4%)	18 (1.6%)	21.2 (1.8%)
**Occupation**					
Unemployed	19,242	3,048 (15.8%)	396 (2.1%)	186 (1%)	236.8 (1.2%)
Occasional/seasonal employment	1,454	250 (17.2%)	35 (2.4%)	19 (1.3%)	22.8 (1.6%)
Employed by government	809	134 (16.6%)	5 (0.6%)	6 (0.7%)	6.2 (0.8%)
Employed in private sector	4,961	815 (16.4%)	74 (1.5%)	38 (0.8%)	47.3 (1%)
Self-employed	7,226	1,564 (21.6%)	68 (0.9%)	39 (0.5%)	47.8 (0.7%)

Pupil / student	6,450	118 (1.8%)	27 (0.4%)	11 (0.2%)	13.9 (0.2%)
Housewife / homemaker	2,629	502 (19.1%)	23 (0.9%)	3 (0.1%)	5.5 (0.2%)
Working on own land	178	23 (12.9%)	1 (0.6%)	0 (0%)	-
Other	668	158 (23.7%)	11 (1.6%)	0 (0%)	-
**Crowding index†**					
Mean (SD)	1.72	-	1.72(1.2)	1.73(1.1)	-
**Smoking status**					
Never smoked	31,957	5,119 (16%)	345 (1.1%)	102 (0.3%)	141.9 (0.4%)
Current smoker	9,425	1,145 (12.1%)	239 (2.5%)	180 (1.9%)	212.9 (2.3%)
Past smoker	2,235	348 (15.6%)	56 (2.5%)	20 (0.9%)	26.3 (1.2%)
Main exposure (HIV status)					
Newly diagnosed, HIV-negative	37,005	-	522 (1.4%)	236 (0.6%)	294.6 (0.8%)
PLHIV, on ART	6,612	-	118 (1.8%)	66 (1.0%)	86.5 (1.3%)

Note: ‡ Socio-economic status = Estimated from household asset ownership (separately for Zambia and SA) using Principal Components Analysis, with the first principal component categorized into quintiles. Further details in the appendix (Text A). § Alcohol use disorder = Scored and classified according to the WHO AUDIT screening tool (Saunders et al., 1993). Further details in the appendix (Text B). †Crowding index = number of people per room in a household. Further details on the distribution are in the appendix (Text C).SD = Standard deviation. %Row percentages

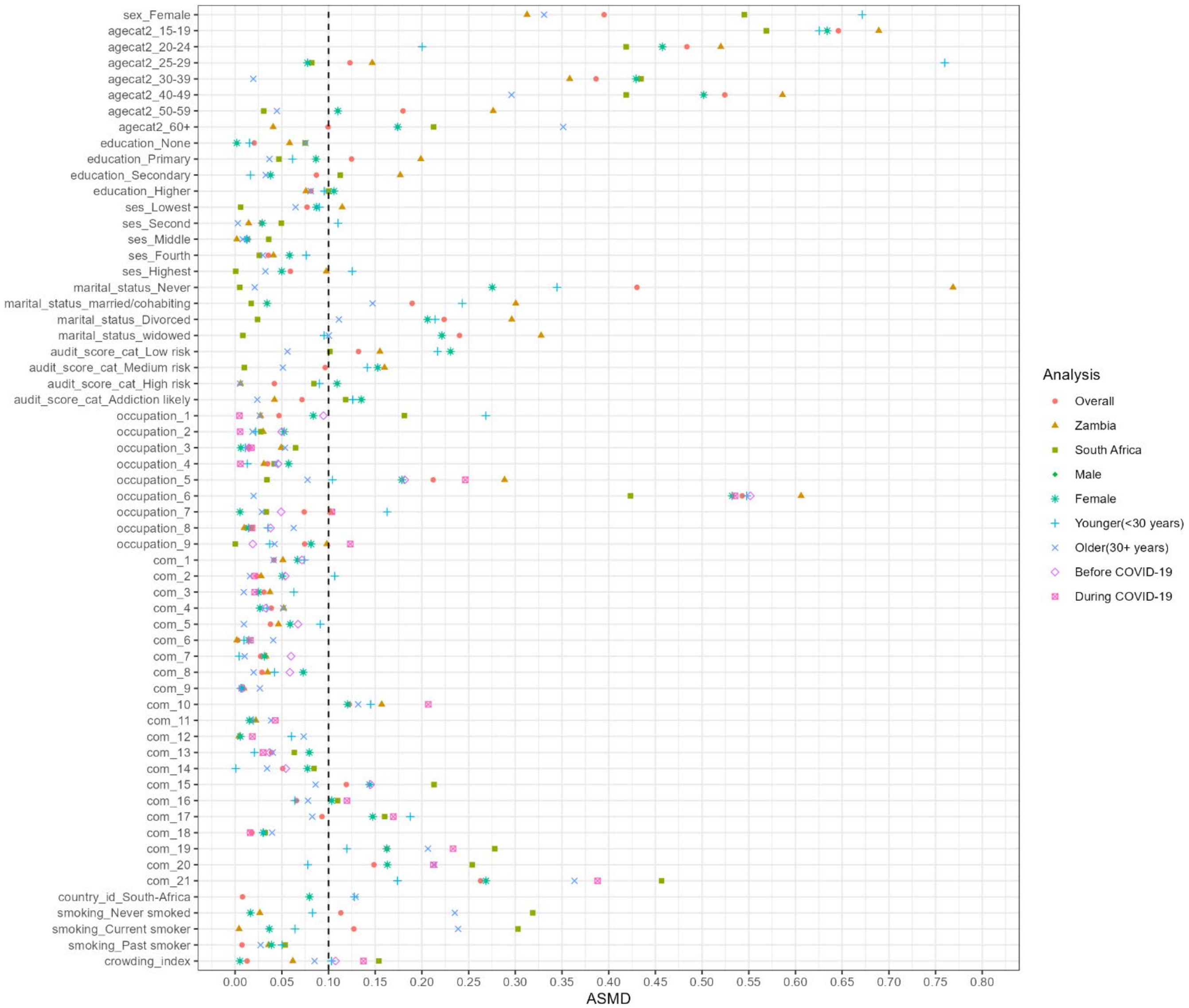

Fig I: Covariate balance before weighting.

The absolute standardized mean differences before (unadjusted) for all individuals' characteristics included in the propensity score model. Characteristics with values <0.1 (i.e. to the left of the dashed line), show good covariate balance.

Note: Occupation_1=Unemployed; Occupation_2=Occasional/seasonal employment; Occupation_3=Employed by government; Occupation_4=Employed in private sector; Occupation_5=Self-employed; Occupation_6=Pupil / student; Occupation_7=Housewife / homemaker; Occupation_8=Working on own land; Occupation_9=Other

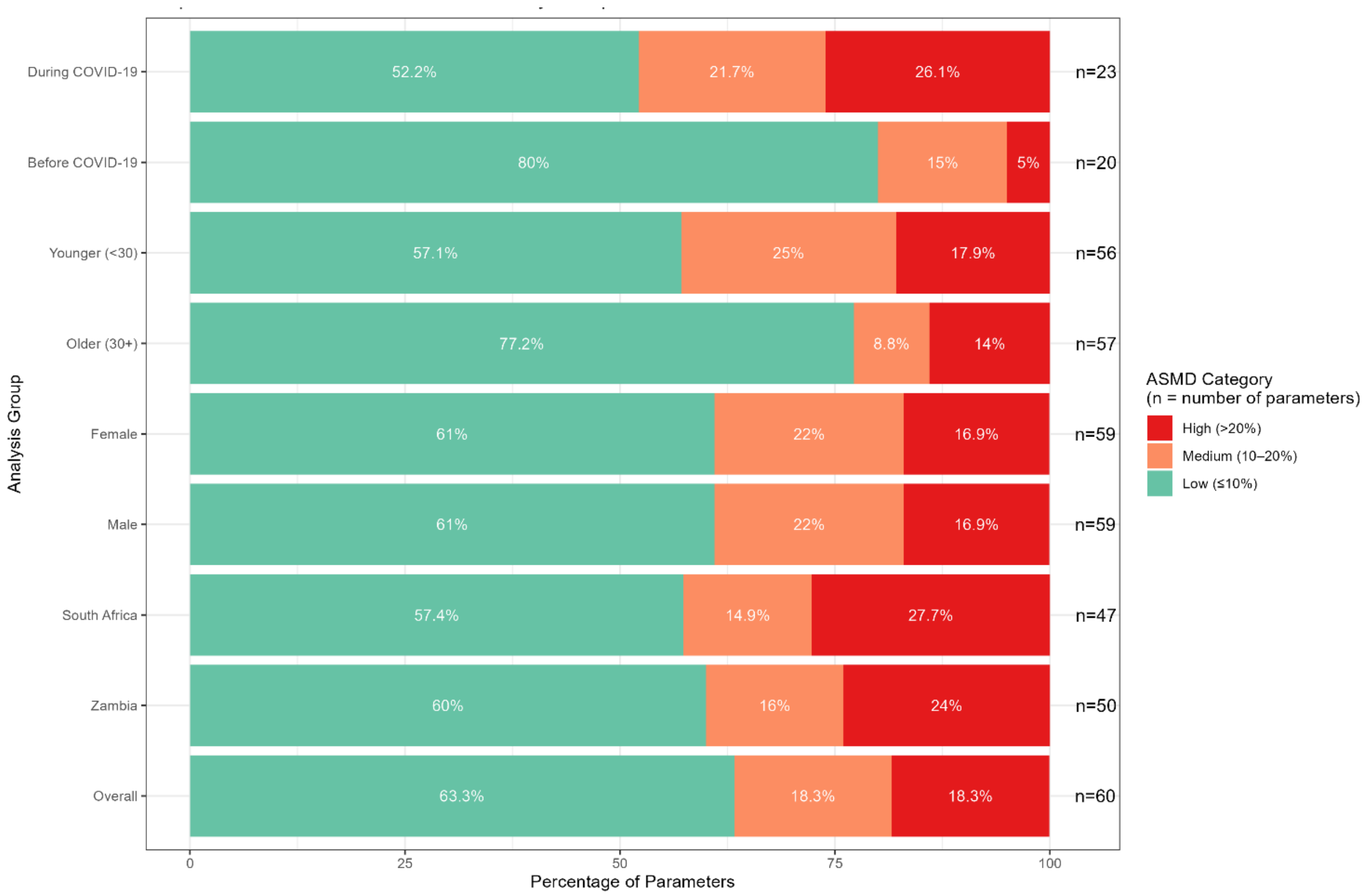

Fig J: Proportion of parameters with initial covariate imbalance, categorized by ASMD level

Note: ASMD = Absolute standardized mean difference; n = Number of coefficients of the covariates adjusted for in each analysis group

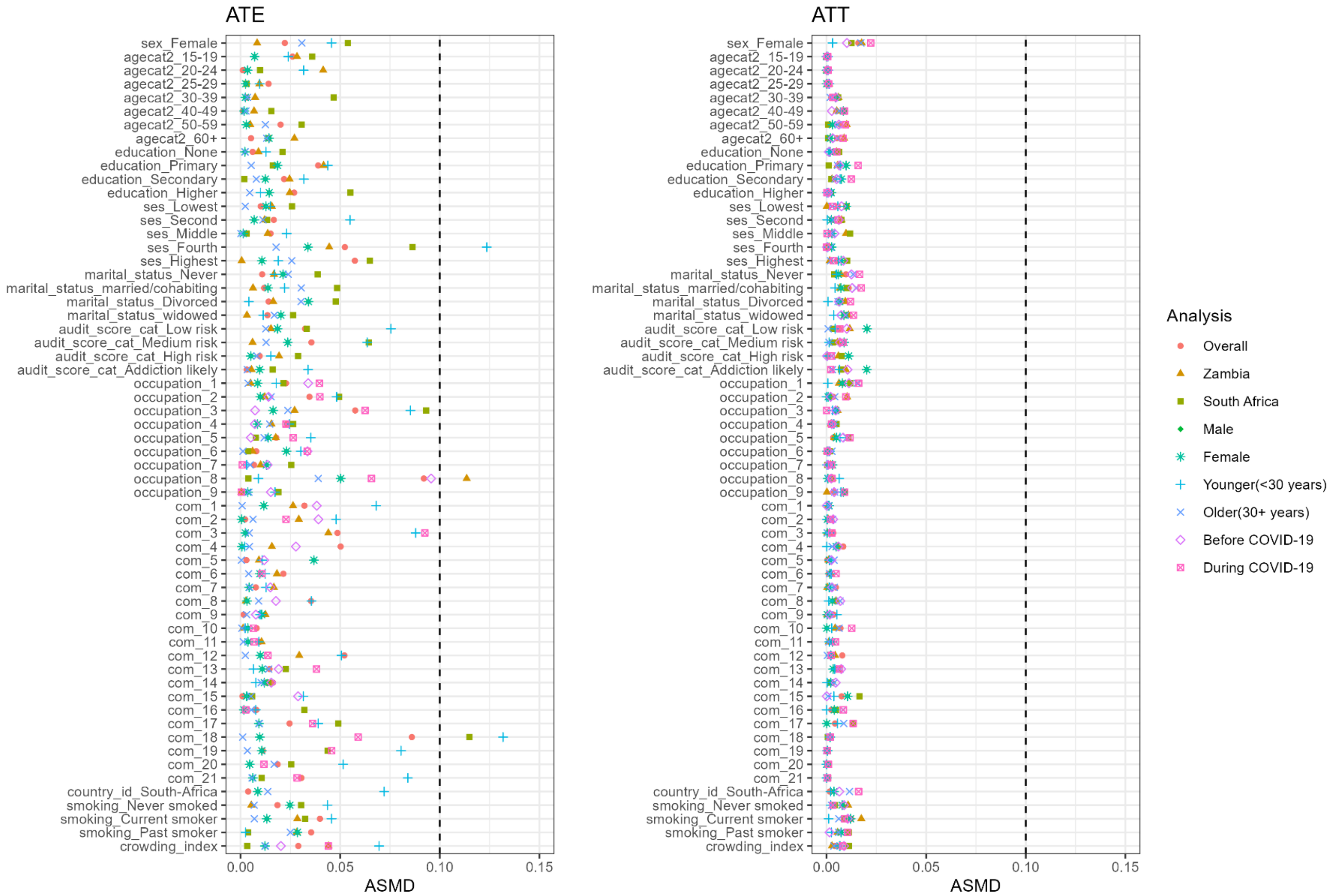

Fig K: Covariate balance after weighting

Note: Occupation_1=Unemployed; Occupation_2=Occasional/seasonal employment; Occupation_3=Employed by government; Occupation_4=Employed in private sector; Occupation_5=Self-employed; Occupation_6=Pupil / student; Occupation_7=Housewife / homemaker; Occupation_8=Working on own land; Occupation_9=Other

Table C: Logistic regression model fitted to estimate the overall propensity score

Potential Confounders	AOR (95%CI)	P-value
**Country**		
Zambia	Reference	-
South Africa	0.55(0.37,0.82)	0.003
**Sex**		
Male	Reference	-
Female	1.56(1.10,2.20)	0.01
**Sex x Country**		
Female x South Africa	1.49(1.29,1.72)	< 0.001
**Age Category (in years)**		
15-19	Reference	
20-24	0.58(0.36,0.94)	
25-29	2.15(1.44,3.20)	
30-39	10.01(7.07,14.17)	
40-49	27.63(19.5,39.15)	
50-59	28.60(19.86,41.18)	
60+	10.62(7.25,15.58)	< 0.001
**Age x Country**		
20-24 x South Africa	1.71(1.09,2.68)	
25-29 x South Africa	1.44(0.95,2.18)	
30-39 x South Africa	1.31(0.88,1.95)	
40-49 x South Africa	0.89(0.59,1.32)	
50-59 x South Africa	0.63(0.41,0.95)	
60+ x South Africa	0.71(0.44,1.14)	< 0.001
**Sex x Age**		
Female x 20-24	3.27(1.97,5.44)	
Female x 25-29	3.02(1.98,4.60)	
Female x 30-39	1.67(1.16,2.42)	
Female x 40-49	1.05(0.72,1.52)	
Female x 50-59	0.53(0.36,0.78)	
Female x 60+	0.34(0.22,0.52)	< 0.001
**Highest Level of education completed**		
None	Reference	
Primary	1.34(1.14,1.58)	
Secondary	1.37(1.16,1.61)	
Higher	0.91(0.72,1.13)	< 0.001
**Alcohol use disorder**[§]		
Low risk:0-7	Reference	
Medium risk:8-15	1.46(1.33,1.60)	
High risk:16-19	1.53(1.29,1.82)	
Addiction likely:20-40	1.79(1.52,2.11)	< 0.001
**Marital Status**		
Never married	Reference	
Currently married or living as married	0.70(0.65,0.76)	
Divorced or separated	1.06(0.94,1.19)	

Widowed	1.57(1.36,1.80)	< 0.001
**Socio-economic Status‡**		
Lowest	Reference	
Second	0.91(0.84,1.00)	
Middle	0.86(0.78,0.94)	
Fourth	0.83(0.75,0.91)	
Highest	0.84(0.77,0.92)	< 0.001
**Occupation**		
Unemployed	Reference	
Occasional/seasonal employment	0.88(0.75,1.03)	
Employed by government	0.72(0.59,0.89)	
Employed in private sector	0.83(0.76,0.91)	
Self-employed	0.91(0.83,0.99)	
Pupil / student	0.53(0.42,0.66)	
Housewife / homemaker	0.91(0.81,1.04)	
Working on own land	0.52(0.33,0.83)	
Other	1.12(0.91,1.37)	< 0.001
**Smoking status**		
Never smoked	Reference	
Current smoker	0.68(0.63,0.75)	
Past smoker	1.05(0.91,1.20)	< 0.001
**Crowding index†**	1.02(0.99,1.05)	0.2

Note: ‡ Socio-economic status = Estimated from household asset ownership (separately for Zambia and SA) using Principal Components Analysis. Further details in the appendix (Text A). § Alcohol use disorder = Scored and classified according to the WHO AUDIT screening tool (Saunders et al., 1993). Further details in the appendix (Text B). †Crowding index = number of people per room in a household. Further details on the distribution are in the appendix (Text C). AOR = Adjusted odds ratio (adjusted for country, sex, age-category, highest level of education, alcohol use disorder, marital status, occupation, smoking status, crowding index and interactions between country: age-category, country: sex and age-category: sex)

Text E: Assessment of positivity and inverse probability weights

Propensity score distributions demonstrated substantial overlap between exposure groups before and after weighting across all overall and subgroup analyses (Figs L - P), supporting the plausibility of the positivity assumption. ATT weights were highly stable, with 99th percentiles generally close to 1 and maximum weights below 5 across all analyses (Table D; Fig Q), indicating limited practical concerns regarding extreme weighting or sparse support regions.

ATE weights showed greater variability and heavier upper tails than ATT weights (Table D; Fig R), particularly in younger individuals and some country-specific analyses, reflecting the larger extrapolation required when estimating population-average counterfactual contrasts. However, the proportion of observations with extreme weights remained very small across analyses (approximately 1% above the 99th percentile threshold), and weighted propensity score distributions continued to demonstrate substantial overlap between exposure groups. Together, these diagnostics suggest that major violations of positivity were unlikely to materially influence the primary ATT analyses.

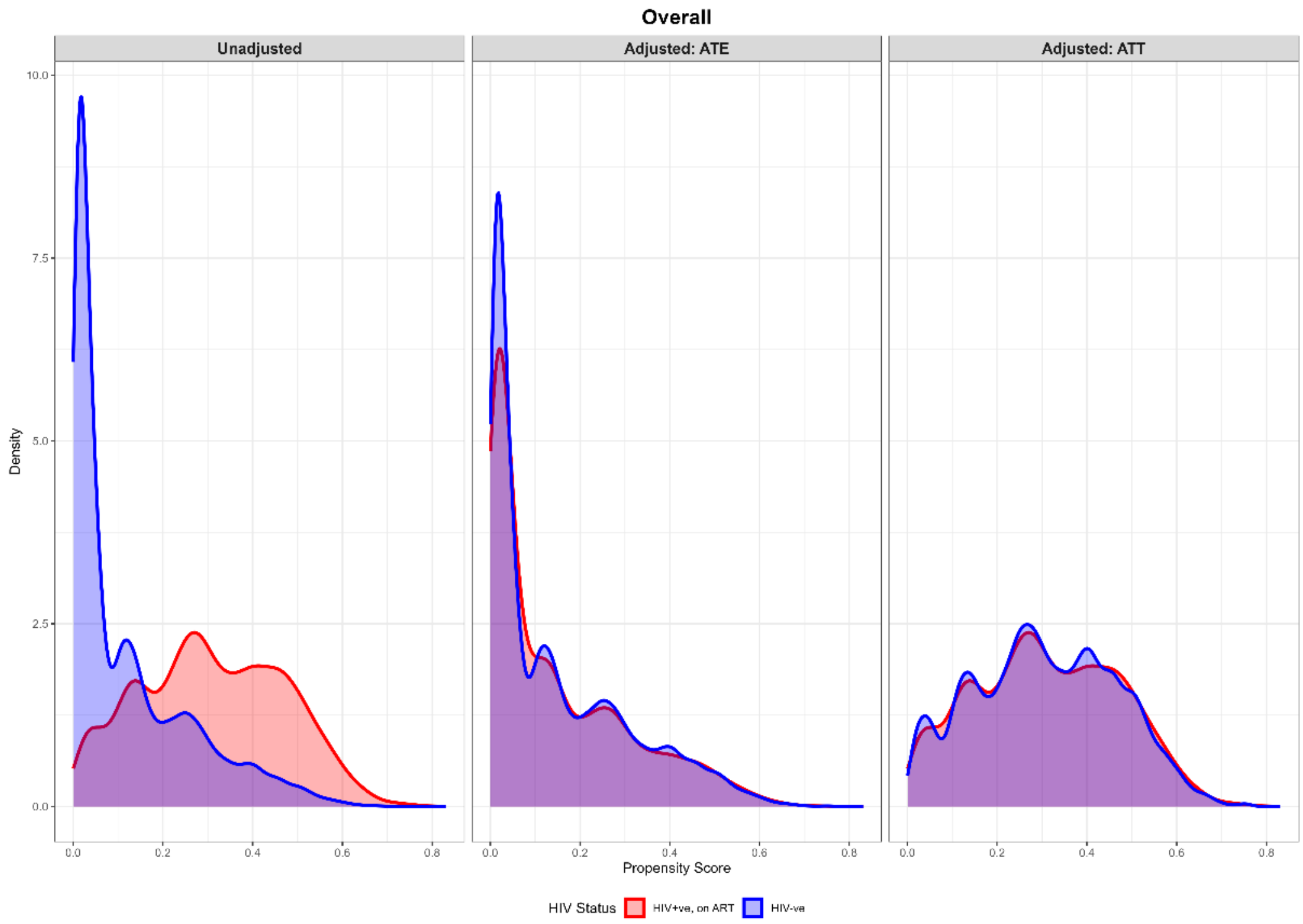

Fig L: Propensity score overlap plot, overall

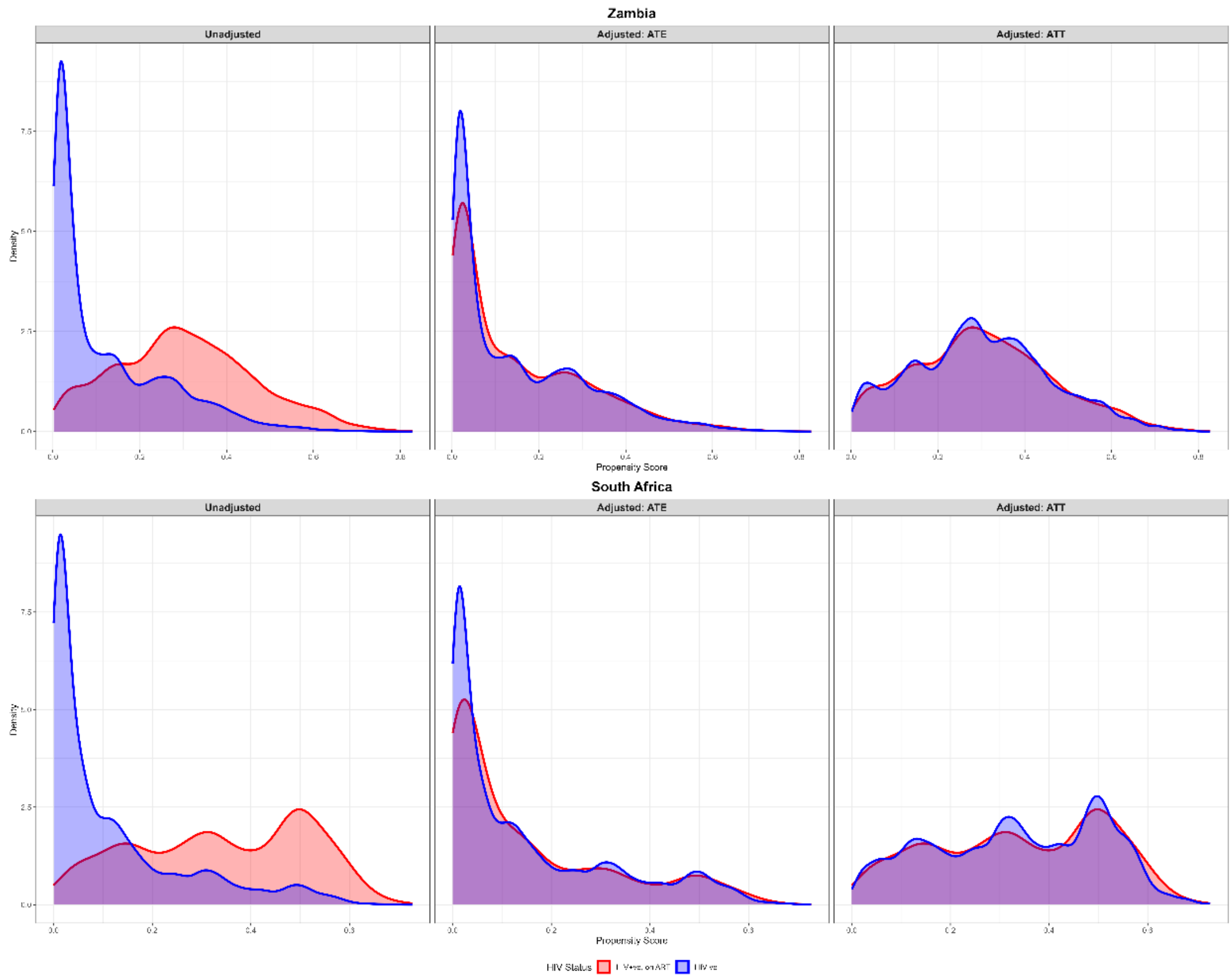

Fig M: Propensity score overlap plot by country

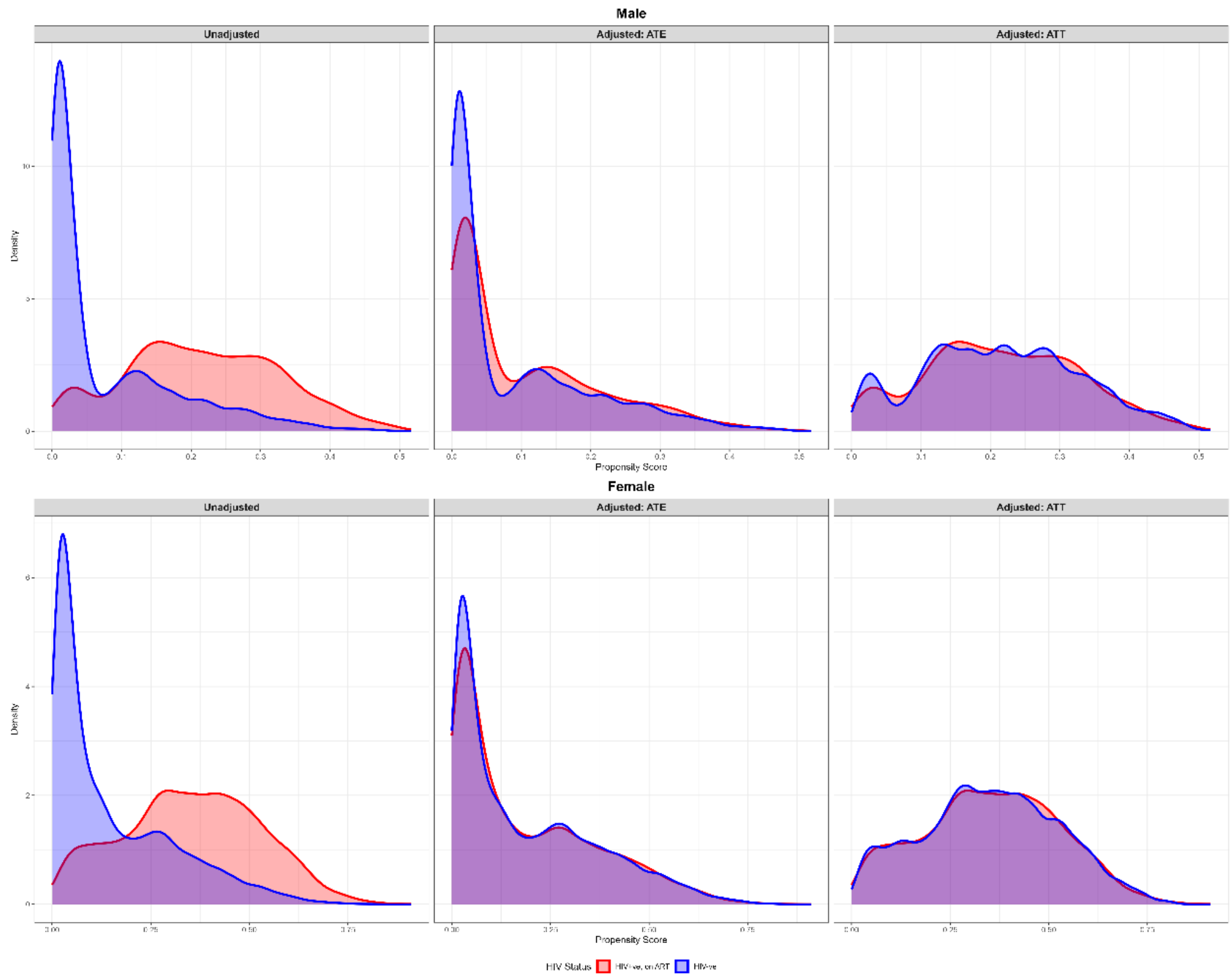

Fig N: Propensity score overlap plot by Sex

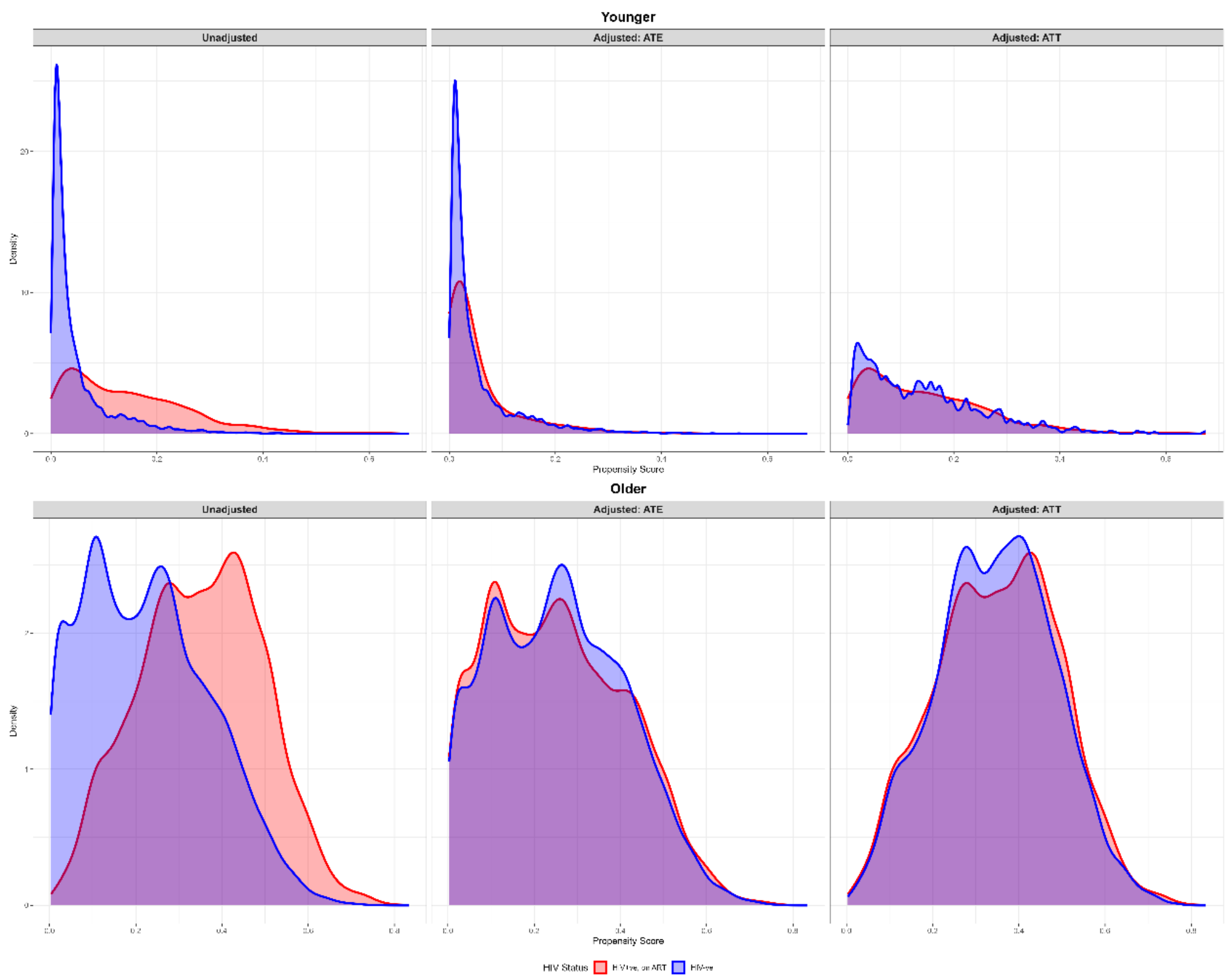

Fig O: Propensity score overlap plot by Age

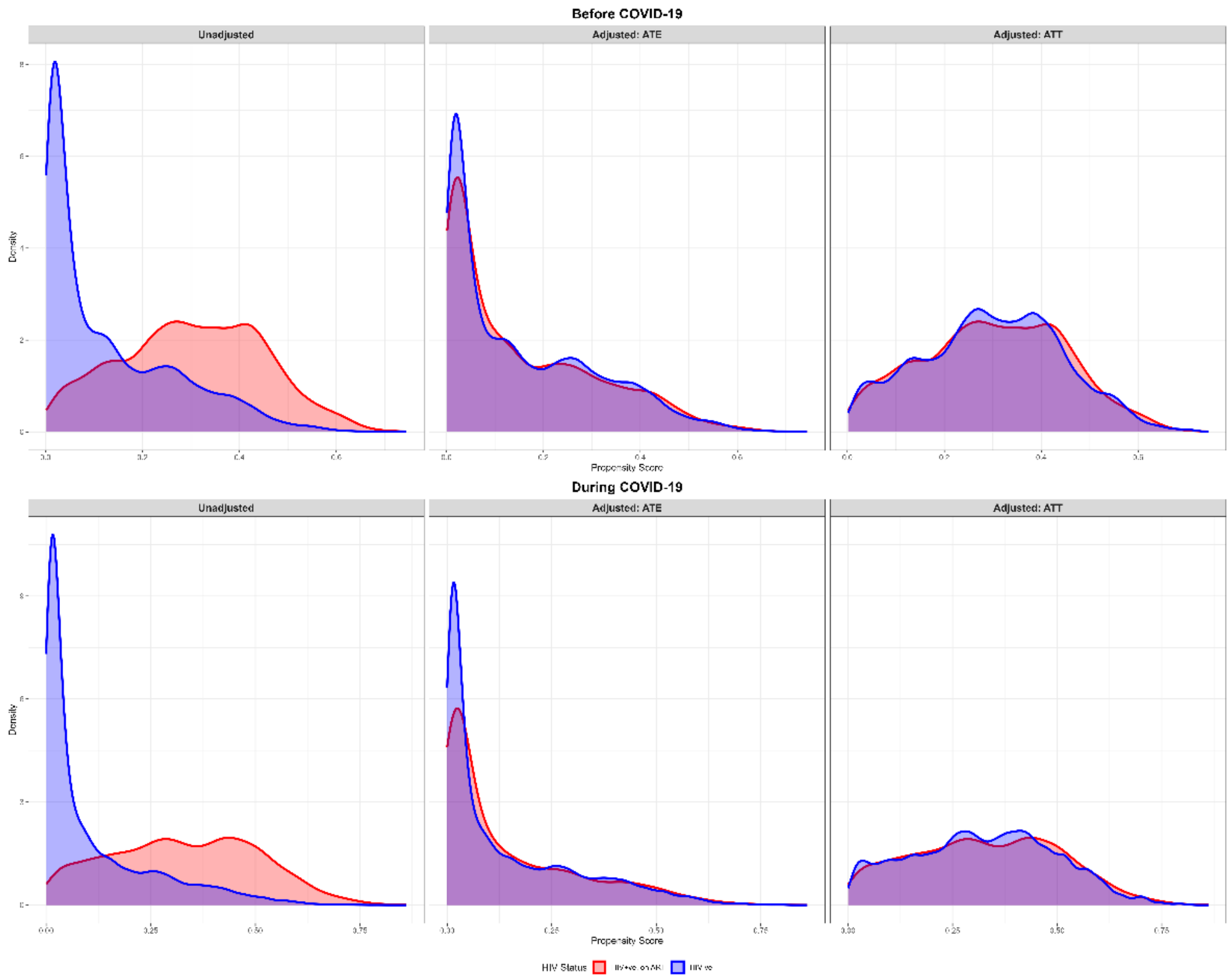

Fig P: Propensity score overlap plot by COVID-19 period

Table D: Summary statistics for the inverse probability weights used in ATT and ATE estimands, overall and separately for each subgroup

Analysis	Weight type*	N	Mean	SD	Min	p1	p5	median	p95	p99	Max	Number above p99 (%)
Overall	ATE	43617	2.021064	5.863471	1.000303	1.002169	1.008032	1.116703	4.277077	16.841755	361.670536	437(1.001903)
Overall	ATT	43617	0.302637	0.374824	0.000303	0.002169	0.008032	0.116703	1.000000	1.097885	3.065272	437(1.001903)
Zambia	ATE	27827	1.993608	5.074416	1.001578	1.005246	1.008929	1.115495	4.293060	16.552574	162.891465	279(1.002623)
Zambia	ATT	27827	0.301319	0.371999	0.001578	0.005246	0.008929	0.115495	1.000000	1.090088	3.398056	279(1.002623)
South Africa	ATE	15790	2.006420	6.627574	1.000000	1.000000	1.003693	1.096821	4.000120	15.960156	395.364863	158(1.000633)
South Africa	ATT	15790	0.304835	0.390999	0.000000	0.000000	0.003693	0.096821	1.000000	1.225122	2.664994	158(1.000633)
Male	ATE	16876	1.883624	5.853821	1.000000	1.000000	1.000000	1.036507	4.414884	15.161044	259.509469	169(1.001422)
Male	ATT	16876	0.182670	0.292494	0.000000	0.000000	0.000000	0.036507	1.000000	1.000000	1.070290	2(0.011851)
Female	ATE	26741	2.002236	4.417647	1.000000	1.004912	1.011001	1.167709	4.183882	16.424190	200.768111	268(1.002206)
Female	ATT	26741	0.379176	0.417920	0.000000	0.004912	0.011001	0.167713	1.000000	1.401300	4.095259	268(1.002206)
Younger	ATE	22727	2.015584	7.520706	1.000000	1.001800	1.004637	1.026196	2.852155	27.437372	240.649305	228(1.003212)
Younger	ATT	22727	0.105724	0.227352	0.000000	0.001800	0.004637	0.026196	1.000000	1.000000	2.076200	6(0.0264)
Older	ATE	20890	2.013512	2.827788	1.002997	1.007414	1.027207	1.404103	4.566583	9.976334	181.314326	209(1.000479)
Older	ATT	20890	0.516003	0.384929	0.002997	0.007414	0.027207	0.406028	1.000000	1.254271	3.151546	209(1.000479)
Before COVID	ATE	23269	2.019184	5.474379	1.001380	1.004020	1.007742	1.135137	4.436698	15.422711	201.805166	233(1.001332)
Before COVID	ATT	23269	0.316395	0.369206	0.001380	0.004020	0.007742	0.135137	1.000000	1.012242	2.470632	233(1.001332)
During COVID	ATE	20348	1.987864	5.643869	1.000000	1.000000	1.005686	1.077494	3.983162	17.864213	175.710433	204(1.002556)
During COVID	ATT	20348	0.286188	0.381665	0.000000	0.000000	0.005686	0.077494	1.000000	1.164508	3.837906	204(1.002556)

Note: *For ATT estimation, treated individuals received weight = 1 and untreated individuals were weighted by PS/(1−PS). For ATE estimation, treated individuals were weighted by 1/PS and untreated individuals by 1/(1−PS), where PS = propensity score.

Mean = average inverse probability weight; SD = standard deviation of the weights; Min and Max = minimum and maximum observed weights; p1 and p5 = 1st and 5th percentiles; p95 and p99 = 95th and 99th percentiles of the weight distribution; % = Row percentage (i.e. number above p99/N)

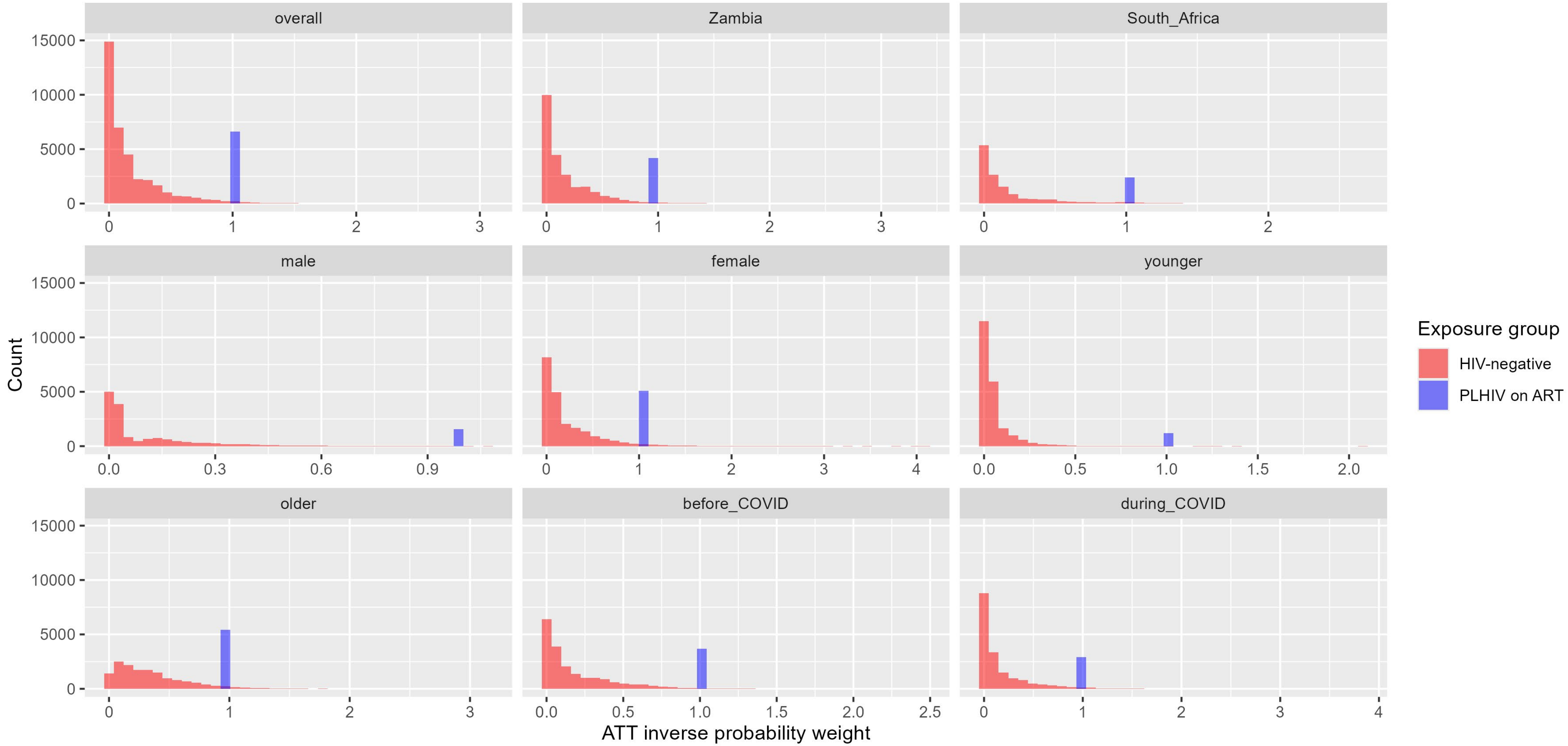

Fig Q: Distribution of ATT weights by exposure group

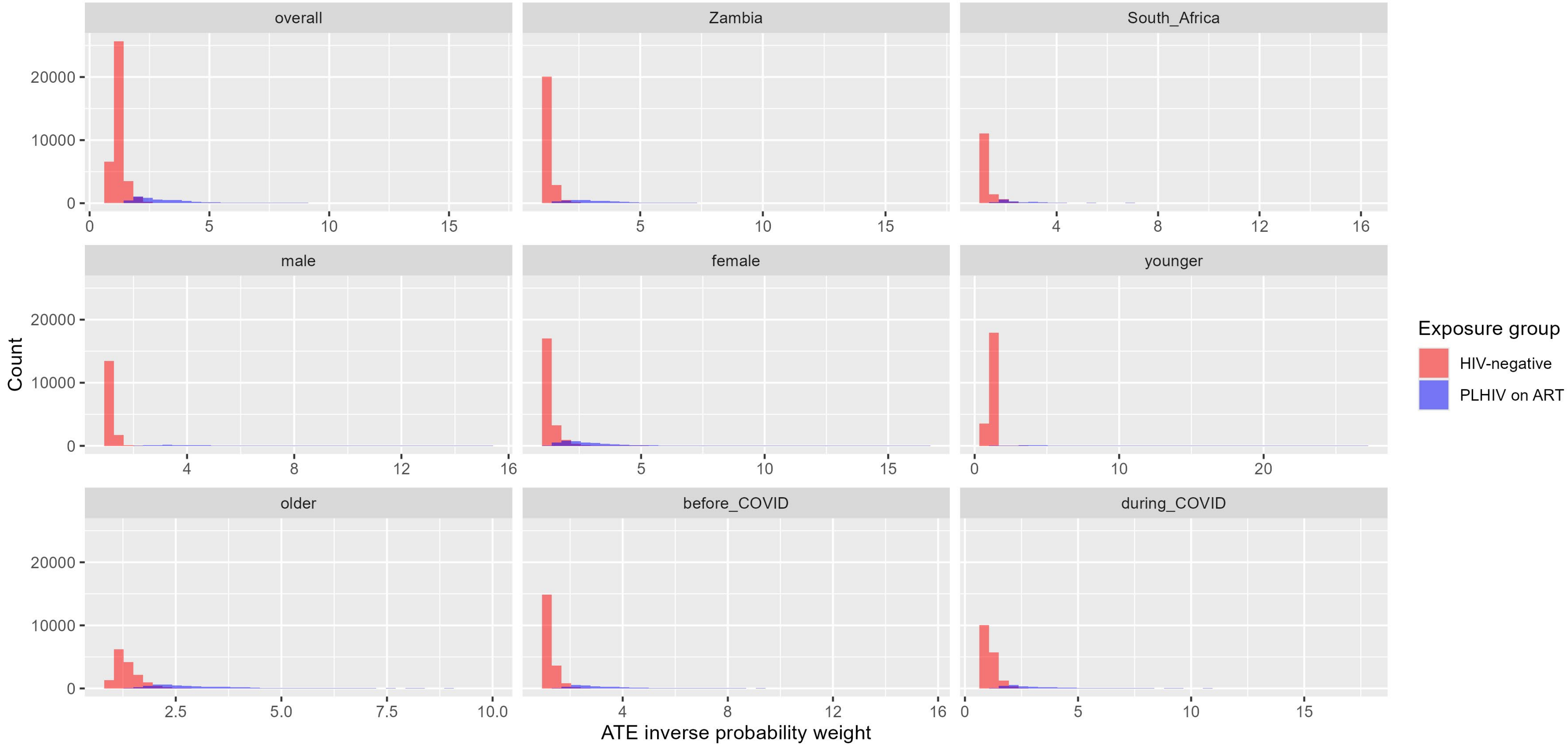

Fig R: Distribution of ATE weights by exposure group

Text F: Comparison of ATE and ATT estimates

ATE and the ATT represent different estimands. The ATE reflects the population-average difference in TB prevalence if the entire study population were hypothetically shifted between exposure states, whereas the ATT estimates (as detailed in the main text) the difference specifically among PLHIV on ART by comparing their observed TB prevalence with the counterfactual prevalence expected if those same individuals had instead been HIV-negative but otherwise similar with respect to measured covariates.

In the overall analysis (Fig S), the ATT estimate was larger than the ATE estimate. The ATE suggested a modest adjusted difference in TB prevalence between PLHIV on ART and HIV-negative individuals (PD = 0.23%, 95% CI −0.30 to 0.76), whereas the ATT indicated a larger difference within the treated population (PD = 0.53%, 95% CI 0.19 to 0.87). A similar pattern was observed across several subgroups. For example, in Zambia the ATT estimate (PD = 0.49%, 95% CI 0.14 to 0.85) exceeded the ATE estimate (PD = 0.38%, 95% CI −0.23 to 1.00). Among females, the ATT (PD = 0.61%, 95% CI 0.27 to 0.95) was larger than the ATE (RD=0.32%, 95% CI −0.01 to 0.66), and among individuals aged ≥30 years, the ATT (PD = 0.52%, 95% CI 0.14 to 0.90) was greater than the ATE (PD = 0.23%, 95% CI −0.20 to 0.66). During the COVID-19 period, the ATT suggested a positive difference (PD = 0.32%, 95% CI −0.14 to 0.79), while the ATE was close to null (PD = −0.32%, 95% CI −0.66 to 0.03).

In other strata, such as SA and among males, the ATE and ATT estimates were closer and had wider confidence intervals.

Interpretation

One plausible explanation for the generally larger ATT estimates is effect heterogeneity. The ATT reflects the difference in TB prevalence specifically among individuals living with HIV and on ART, who may have a higher baseline prevalence of TB due to immunological, environmental, or social factors. In contrast, the ATE averages the effect across the entire study population, including lower-risk HIV-negative individuals, which may attenuate the population-average estimate. If the difference in TB prevalence associated with HIV status is greater among higher-risk individuals, this would lead to systematically larger ATT estimates compared with ATE estimates.

However, these comparisons between ATE and ATT were not part of the primary prespecified analysis and should therefore be interpreted cautiously. The observed differences are descriptive and hypothesis-generating rather than confirmatory. Further work would be needed to formally assess effect heterogeneity, including exploration of interaction effects, alternative model specifications, and assessment of overlap within specific subgroups.

Fig S: ATE and ATT prevalence difference estimates comparing TB prevalence between PLHIV on ART and HIV-negative individuals: overall and by subgroup

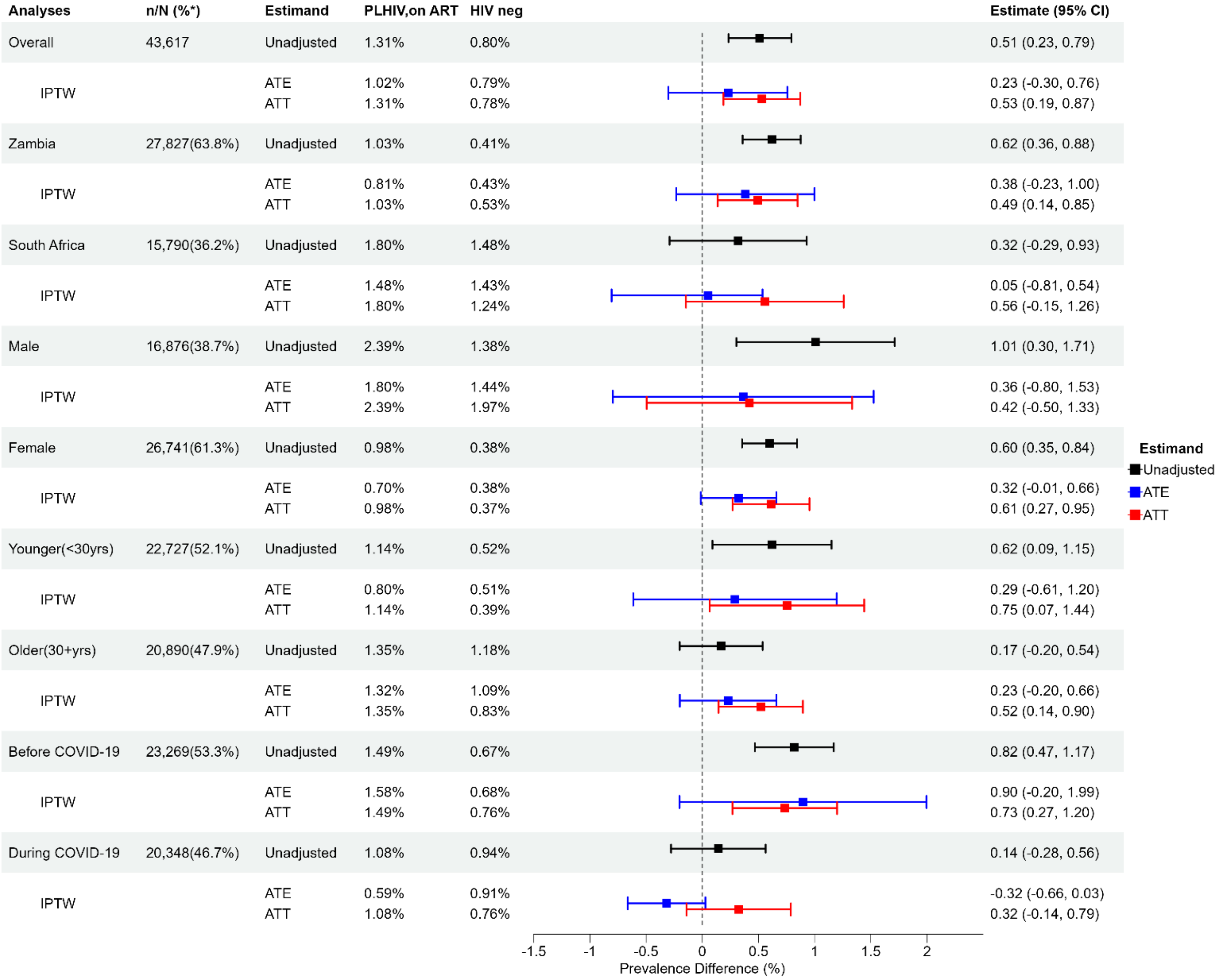

Note: ATE = Average treatment effect, ATT = average treatment effect among the treated, PLHIV = People living with HIV, HIV neg = HIV negative, CI = Confidence interval, n/N%* (N = 43,617), IPTW = Inverse probability of treatment weighting

Text G: Sensitivity analysis

E-values

To assess the robustness of our findings to potential unmeasured confounding, we computed E-values for both the point estimates and the lower confidence interval limits of our primary causal estimates. The E-value quantifies the minimum strength of association that an unmeasured confounder would need to have with both the exposure and outcome, conditional on measured confounders, to fully explain away the observed exposure-outcome association [19–21]. In our case, the E-value represents the minimum risk difference by which an unmeasured confounder would need to be associated with both HIV status and prevalent TB to nullify the observed effect. This metric provides a transparent way to evaluate the plausibility of unmeasured confounding as an alternative explanation for our findings. The E-values were calculated using specialized software, consistent with the methodology outlined by Vanderweele and Ding [20].

Results

For the primary ATT estimate of a 0.53% risk difference (95% CI: 0.19, 0.87), the corresponding E-value was 2.75. This indicates that an unmeasured confounder would need to be associated with both HIV status and prevalent TB with a risk difference of at least 2.75 percentage points, conditional on the measured covariates, to fully explain away the observed association. Given the substantial magnitude of this required association, it is unlikely that a single unmeasured confounder could account for the observed ATT.

Furthermore, to shift the lower bound of the confidence interval (0.19%) to the null, an unmeasured confounder would need to be associated with both the exposure and the outcome with a risk difference of at least 1.92 percentage points. This finding suggests that our conclusion of a significant residual burden of TB among the treated population is robust to unmeasured confounding of a considerable magnitude.

References

1. Howe LD, Hargreaves JR, Huttly SR. Issues in the construction of wealth indices for the measurement of socio-economic position in low-income countries. Emerg Themes Epidemiol. 2008;5: 3. doi:10.1186/1742-7622-5-3

2. Babor TF, Higgins-Biddle JC, Saunders JB, Monteiro MG. AUDIT: The Alcohol Use Disorders Identification Test. Guidelines for Use in Primary Care. Second Edition. World Health Organization: Department of Mental Health and Substance Dependence; Available: https://www.paho.org/sites/default/files/Auditmanual_ENG.pdf

3. Saunders JB, Aasland OG, Babor TF, De La Fuente JR, Grant M. Development of the Alcohol Use Disorders Identification Test (AUDIT): WHO Collaborative Project on Early Detection of Persons with Harmful Alcohol Consumption-II. Addiction. 1993;88: 791–804. doi:10.1111/j.1360-0443.1993.tb02093.x

4. Hargreaves JR, Boccia D, Evans CA, Adato M, Petticrew M, Porter JDH. The Social Determinants of Tuberculosis: From Evidence to Action. Am J Public Health. 2011;101: 654–662. doi:10.2105/AJPH.2010.199505

5. Swartwood NA, Singh N, Mortazavi SA, Can MH, Cui H, Ryuk DK, et al. Differences in tuberculosis prevalence by sex in low- and middle-income countries over 1993–2025: A systematic review and meta-analysis. Tang S, editor. PLOS Med. 2026;23: e1005114. doi:10.1371/journal.pmed.1005114

6. Tuot S, Sim JW, Nagashima-Hayashi M, Chhoun P, Teo AKJ, Prem K, et al. What are the determinants of antiretroviral therapy adherence among stable people living with HIV? A cross-sectional study in Cambodia. AIDS Res Ther. 2023;20: 47. doi:10.1186/s12981-023-00544-w

7. Heestermans T, Browne JL, Aitken SC, Vervoort SC, Klipstein-Grobusch K. Determinants of adherence to antiretroviral therapy among HIV-positive adults in sub-Saharan Africa: a systematic review. BMJ Glob Health. 2016;1: e000125. doi:10.1136/bmjgh-2016-000125

8. Dyson T. HIV/AIDS and Urbanization. Popul Dev Rev. 2003;29: 427–442. doi:10.1111/j.1728-4457.2003.00427.x

9. Choi S-W, Im J-J, Yoon S-E, Kim S-H, Cho J-H, Jeong S-J, et al. Lower socioeconomic status associated with higher tuberculosis rate in South Korea. BMC Pulm Med. 2023;23: 418. doi:10.1186/s12890-023-02713-z

10. Lönnroth K, Jaramillo E, Williams BG, Dye C, Raviglione M. Drivers of tuberculosis epidemics: The role of risk factors and social determinants. Soc Sci Med. 2009;68: 2240–2246. doi:10.1016/j.socscimed.2009.03.041

11. Burch LS, Smith CJ, Anderson J, Sherr L, Rodger AJ, O'Connell R, et al. Socioeconomic status and treatment outcomes for individuals with HIV on antiretroviral treatment in the UK: cross-sectional and longitudinal analyses. Lancet Public Health. 2016;1: e26–e36. doi:10.1016/S2468-2667(16)30002-0

12. Shisana O, Risher K, Celentano DD, Zungu N, Rehle T, Ngcaweni B, et al. Does marital status matter in an HIV hyperendemic country? Findings from the 2012 South African National HIV Prevalence, Incidence and Behaviour Survey. AIDS Care. 2016;28: 234–241. doi:10.1080/09540121.2015.1080790

13. Johnson MO, Dilworth SE, Taylor JM, Darbes LA, Comfort ML, Neilands TB. Primary Relationships, HIV Treatment Adherence, and Virologic Control. AIDS Behav. 2012;16: 1511–1521. doi:10.1007/s10461-011-0021-0

14. Crampin A, Kasimba S, Mwaungulu NJ, Dacombe R, Floyd S, Glynn JR, et al. Married to *M. tuberculosis* : risk of infection and disease in spouses of smear-positive tuberculosis patients. Trop Med Int Health. 2011;16: 811–818. doi:10.1111/j.1365-3156.2011.02763.x

15. Brookhart MA, Schneeweiss S, Rothman KJ, Glynn RJ, Avorn J, Stürmer T. Variable selection for propensity score models. Am J Epidemiol. 2006;163: 1149–1156.

16. Rubin DB. The design versus the analysis of observational studies for causal effects: parallels with the design of randomized trials. Stat Med. 2007;26: 20–36.

17. Austin PC. An Introduction to Propensity Score Methods for Reducing the Effects of Confounding in Observational Studies. Multivar Behav Res. 2011;46: 399–424. doi:10.1080/00273171.2011.568786

18. Ali MS, Groenwold RHH, Pestman WR, Belitser SV, Roes KCB, Hoes AW, et al. Propensity score balance measures in pharmacoepidemiology: a simulation study. Pharmacoepidemiol Drug Saf. 2014;23: 802–811. doi:10.1002/pds.3574

19. Ding P, VanderWeele TJ. Sensitivity Analysis Without Assumptions: Epidemiology. 2016;27: 368–377. doi:10.1097/EDE.0000000000000457

20. VanderWeele TJ, Ding P. Sensitivity Analysis in Observational Research: Introducing the E-Value. Ann Intern Med. 2017;167: 268–274. doi:10.7326/M16-2607

21. Mathur MB, Ding P, Riddell CA, VanderWeele TJ. Web Site and R Package for Computing E-values. Epidemiology. 2018;29: e45–e47. doi:10.1097/EDE.0000000000000864
