# Supplementary material for: Quantifying excess TB prevalence among people living with HIV on ART in the universal ART era: Evidence from a TB prevalence survey in Zambia and South Africa
Source: PLOS Glob Public Health. 2026 Sep 21;6(9):e0006833. doi: 10.1371/journal.pgph.0006833 (PMC13592586; doi:10.1371/journal.pgph.0006833)
Supplement: Supplementary file 2 [file pgph.0006833.s001.doc]

STROBE Statement—checklist of items that should be included in reports of observational studies

|  | Item No | Recommendation |
| --- | --- | --- |
| **Title and abstract** | 1 | (*a*) Indicate the study’s design with a commonly used term in the title or the abstract  [The first sentence of the methods section explicitly says that, ”… Tuberculosis Reduction through Expanded Anti-retroviral Treatment and Screening (TREATS) TB prevalence survey, a cross-sectional survey undertaken in 21 communities in Zambia and the Western Cape province of South Africa (SA),...”] |
|  |  | (*b*) Provide in the abstract an informative and balanced summary of what was done and what was found  [The abstract provides a structured and balanced summary of the study. The Background outlines the scientific problem and rationale; the Methods describe the study design, exposure definition, estimand (ATT), causal framework and statistical approach; the Results report participant numbers, prevalence estimates and effect estimates; and the Conclusion summarizes the implications of the findings.] |
| Introduction | | |
| Background/rationale | 2 | Explain the scientific background and rationale for the investigation being reported  [The scientific background and rationale are presented in the Introduction (paragraphs 1–5). Specifically: Paragraphs 1–2 describe the global burden of TB and HIV and the importance of ART. Paragraph 3 explains why TB prevalence remains important despite reductions in TB incidence. Paragraph 4 discusses the TREATS survey and limitations of previous analyses. Paragraph 5 explains the need for a causal inference framework and prevalence differences as the target measure.] |
| Objectives | 3 | State specific objectives, including any prespecified hypotheses  [See the end of the “Introduction” section, paragraph 6 i.e. “In the analyses presented here, we applied the Rubin’s potential outcomes framework to estimate the excess prevalence of TB within the population of PLHIV who were on ART.”] |
| Methods | | |
| Study design | 4 | Present key elements of study design early in the paper  [Provided in the” Materials and Methods” section under “Study design and setting” subsection describes the key design elements: secondary causal analysis of the TREATS cross-sectional TB prevalence survey using Rubin’s potential outcomes framework and IPTW] |
| Setting | 5 | Describe the setting, locations, and relevant dates, including periods of recruitment, exposure, follow-up, and data collection  [Provided in the” Materials and Methods” section under “Study design and setting” the setting, countries (Zambia and Western Cape, South Africa), communities (21 total), and the exact data collection dates (between 2019–2021, including COVID-19 period) are clearly described] |
| Participants | 6 | (*a*) *Cohort study*—Give the eligibility criteria, and the sources and methods of selection of participants. Describe methods of follow-up  *Case-control study*—Give the eligibility criteria, and the sources and methods of case ascertainment and control selection. Give the rationale for the choice of cases and controls  *Cross-sectional study*—Give the eligibility criteria, and the sources and methods of selection of participants  [In the “Material and Methods” section eligible participants were randomly sampled individuals aged ≥15 years attending mobile field sites under the “Study design and setting”. Inclusion and exclusion criteria (new HIV diagnoses, PLHIV not on ART, unknown status) are explicitly reported under “Exposure definition and comparison group” subsection. Methods of recruitment and TB screening are described.] |
|  |  | (*b*) *Cohort study*—For matched studies, give matching criteria and number of exposed and unexposed  *Case-control study*—For matched studies, give matching criteria and the number of controls per case |
| Variables | 7 | Clearly define all outcomes, exposures, predictors, potential confounders, and effect modifiers. Give diagnostic criteria, if applicable  [The outcome (bacteriologically confirmed prevalent TB), exposure (HIV/ART status), and confounders identified via DAG (age, sex, country, social economic status [SES], education, marital status, occupation, alcohol use disorder, smoking, crowding index) are clearly defined] |
| Data sources/ measurement | 8* | For each variable of interest, give sources of data and details of methods of assessment (measurement). Describe comparability of assessment methods if there is more than one group  [Detailed measurement methods are provided, including:  a] Questionnaires for socio-demographic and behavioural data  b] Digital chest X-ray with CAD scoring  c] Xpert-Ultra and culture for TB diagnosis  d] PCA for SES  e] WHO AUDIT for alcohol use disorder  Comparability across groups is inherent as the same survey procedures were applied.] |
| Bias | 9 | Describe any efforts to address potential sources of bias  [Several approaches were used to minimise bias.  a] A directed acyclic graph was used to identify a minimally sufficient confounder adjustment set.  b] Propensity scores were estimated and used to generate inverse probability of treatment weights.  c] Positivity was evaluated through propensity score overlap, while covariate balance was assessed using standardised mean differences and love plots.  Remaining sources of potential bias, including self-reported ART use, residual confounding, survival bias, cross-sectional temporality, and interference, are discussed in the Discussion section.] |
| Study size | 10 | Explain how the study size was arrived at  [The sample size (~50,000 target; 43,617 analysed) was determined by the parent TREATS TB prevalence survey design. No additional power calculation was performed for this secondary analysis] |
| Quantitative variables | 11 | Explain how quantitative variables were handled in the analyses. If applicable, describe which groupings were chosen and why  [Continuous variables were categorised a priori where appropriate (e.g., age groups, SES quintiles, AUDIT categories).  Prevalence differences were estimated on the absolute scale for interpretability.] |
| Statistical methods | 12 | (*a*) Describe all statistical methods, including those used to control for confounding  [Propensity scores were estimated using logistic regression including prespecified confounders and interaction terms. IPTW was applied to estimate ATT and ATE. Balance was assessed using ASMD (<10% threshold).] |
|  |  | (*b*) Describe any methods used to examine subgroups and interactions  [Prespecified subgroup analyses were conducted by country, sex, age group, and COVID-19 period] |
|  |  | (*c*) Explain how missing data were addressed  [Missing outcome data were handled using multiple imputation (300 datasets) with Rubin’s rules used for pooling estimates. Exact steps of this have been detailed in the primary outcome analysis manuscript (Klinkenberg E & Floyd S, et al., 2023) under the supplementary materials (S1 SAP and S2 Text)] |
|  |  | (*d*) *Cohort study*—If applicable, explain how loss to follow-up was addressed  *Case-control study*—If applicable, explain how matching of cases and controls was addressed  *Cross-sectional study*—If applicable, describe analytical methods taking account of sampling strategy  [The cross-sectional random sampling design of the TREATS survey is described, and IPTW created a pseudo-population balanced on measured confounders] |
|  |  | (*e*) Describe any sensitivity analyses  [Sensitivity to unmeasured confounding was assessed using E-values, and causal assumptions (positivity, balance) were evaluated diagnostically.] |

Continued on next page

| Results | | |
| --- | --- | --- |
| Participants | 13* | (a) Report numbers of individuals at each stage of study—eg numbers potentially eligible, examined for eligibility, confirmed eligible, included in the study, completing follow-up, and analysed  [Participant flow from households to analytic sample is reported numerically and in Fig 2.] |
|  |  | (b) Give reasons for non-participation at each stage  [Reasons for exclusion (new HIV diagnosis, not on ART, unknown status) are provided.] |
|  |  | (c) Consider use of a flow diagram  [A study flow diagram is presented (Fig 2)] |
| Descriptive data | 14* | (a) Give characteristics of study participants (eg demographic, clinical, social) and information on exposures and potential confounders  [Detailed baseline characteristics stratified by HIV/ART status are shown in Table 1.] |
|  |  | (b) Indicate number of participants with missing data for each variable of interest  [Missing TB outcome data were addressed through multiple imputation; prevalence estimates after imputation are reported.] |
|  |  | (c) *Cohort study*—Summarise follow-up time (eg, average and total amount)  [Not applicable (cross-sectional study).] |
| Outcome data | 15* | *Cohort study*—Report numbers of outcome events or summary measures over time |
|  |  | *Case-control study—*Report numbers in each exposure category, or summary measures of exposure |
|  |  | *Cross-sectional study—*Report numbers of outcome events or summary measures  [Overall and subgroup TB prevalence estimates are reported using imputed data.] |
| Main results | 16 | (*a*) Give unadjusted estimates and, if applicable, confounder-adjusted estimates and their precision (eg, 95% confidence interval). Make clear which confounders were adjusted for and why they were included  [Both unadjusted and IPTW-adjusted ATT and ATE risk differences with 95% CIs are reported, with confounders prespecified via DAG.] |
|  |  | (*b*) Report category boundaries when continuous variables were categorized  [All categorical cut-points (e.g., age bands, SES quintiles) are defined in Methods.] |
|  |  | (*c*) If relevant, consider translating estimates of relative risk into absolute risk for a meaningful time period  [Population-level excess TB prevalence attributable to PLHIV on ART is calculated and presented (Table 3).] |
| Other analyses | 17 | Report other analyses done—eg analyses of subgroups and interactions, and sensitivity analyses  [Subgroup ATT estimates and ATE analyses are reported and interpreted.] |
| Discussion | | |
| Key results | 18 | Summarise key results with reference to study objectives  [The opening paragraphs of the Discussion summarise the principal findings and directly relate them to the study objective of estimating excess TB prevalence among PLHIV on ART.] |
| Limitations | 19 | Discuss limitations of the study, taking into account sources of potential bias or imprecision. Discuss both direction and magnitude of any potential bias  [Addressed in the Discussion (paragraph 6).  The Discussion describes limitations including exposure misclassification from self-reported ART use, heterogeneity in ART duration and adherence, cross-sectional temporality, survival bias, residual confounding, measurement error, and interference.] |
| Interpretation | 20 | Give a cautious overall interpretation of results considering objectives, limitations, multiplicity of analyses, results from similar studies, and other relevant evidence  [In paragraph 2-5, findings are cautiously interpreted in light of prior TREATS analyses and other prevalence surveys.] |
| Generalisability | 21 | Discuss the generalisability (external validity) of the study results  [External validity is discussed through comparison of Zambia vs South Africa contexts and implications for high HIV-burden settings] |
| Other information | | |
| Funding | 22 | Give the source of funding and the role of the funders for the present study and, if applicable, for the original study on which the present article is based  [A statement on this was provided as part of the manuscript submission] |

*Give information separately for cases and controls in case-control studies and, if applicable, for exposed and unexposed groups in cohort and cross-sectional studies.

**Note:** An Explanation and Elaboration article discusses each checklist item and gives methodological background and published examples of transparent reporting. The STROBE checklist is best used in conjunction with this article (freely available on the Web sites of PLoS Medicine at http://www.plosmedicine.org/, Annals of Internal Medicine at http://www.annals.org/, and Epidemiology at http://www.epidem.com/). Information on the STROBE Initiative is available at www.strobe-statement.org.
